# ﻿A new species of alkali-sink *Paruroctonus* Werner, 1934 (Scorpiones, Vaejovidae) from California’s San Joaquin Valley

**DOI:** 10.3897/zookeys.1185.103574

**Published:** 2023-11-29

**Authors:** Prakrit Jain, Harper Forbes, Jacob A. Gorneau, Lauren A. Esposito

**Affiliations:** 1 California Academy of Sciences, Institute for Biodiversity Science and Sustainability, 55 Music Concourse Drive, San Francisco, California 94118, USA California Academy of Sciences, Institute for Biodiversity Science and Sustainability San Francisco United States of America

**Keywords:** Central Valley, CITES, desert, salt flats, threatened

## Abstract

Herein a new species of *Paruroctonus* Werner, 1934 is described from alkali-sink habitats in the San Joaquin Desert of central California, *Paruroctonustulare***sp. nov.** It can be differentiated from other *Paruroctonus* by a combination of morphological features including scalloped pedipalp fingers in males, specific setal counts and morphometric ratios, and specific patterns of fuscous pigmentation. It also inhabits a unique distribution allopatric with all other *Paruroctonus* species except *P.variabilis* Hjelle, 1982. Photographs of a large series of live *P.tulare***sp. nov.** from across their range and detailed images of several morphological features are provided, their distribution is modeled, a haplotype network is presented, and details about their habitat, ecology, and conservation are discussed.

## ﻿Introduction

Scorpions in the genus *Paruroctonus* Werner, 1934 exhibit endemism in alkali-sink environments associated with wetlands or dry lake beds in desert regions (Jain et al. 2022). Examples include *Paruroctonusbantaibantai* Gertsch & Soleglad, 1966 from Saline Valley in Death Valley National Park (California), *Paruroctonusbantaisaratoga* Haradon, 1985 from Saratoga Springs in Death Valley National Park, *Paruroctonusconclusus* Jain, Forbes, & Esposito, 2022 from Koehn Lake in the northwest Mojave Desert (California), and *Paruroctonussoda* Jain, Forbes, & Esposito, 2022 from Soda Lake in the Carrizo Plain (California). Alkali-sink environments are typically located in dry endorheic basins where water flow from surrounding mountains has resulted in clay-rich, saline, and alkaline soils at the low point of the basin (Stoffer 2004; Germano et al. 2011). The unique soils of these regions result in unique plant communities largely dominated by halophytic plants and an array of animal species well suited to areas drier than wetlands but relatively mesic compared to the surrounding landscape (Munz and Keck 1949).

**Figure 1.**
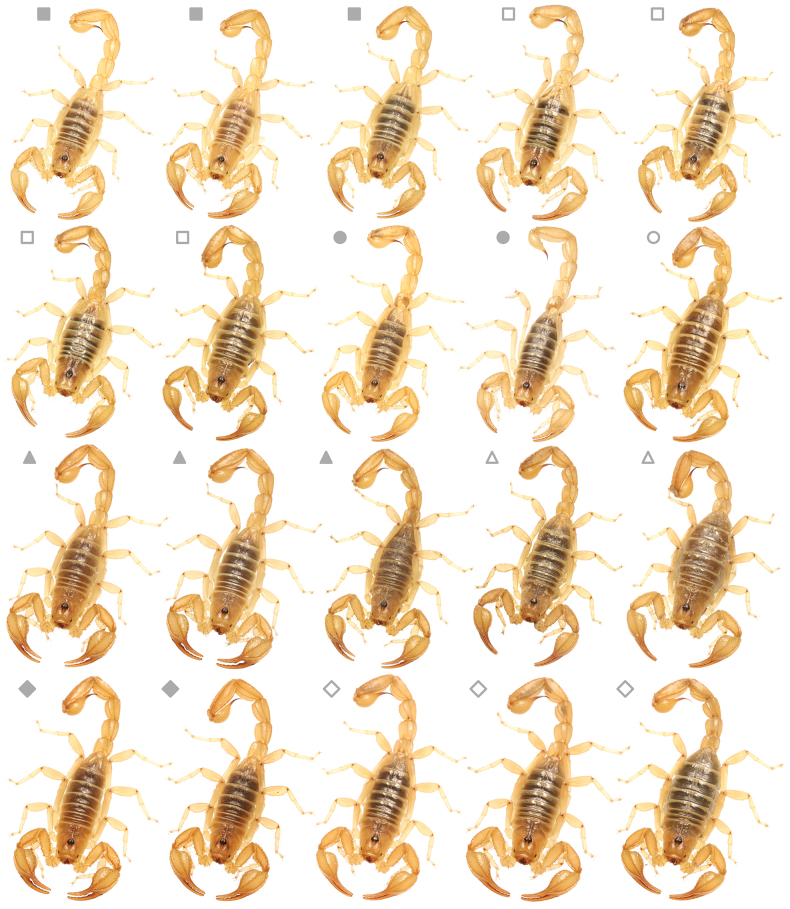
Variation of *Paruroctonustulare* sp. nov. across their geographic range. Central localities C1–9 (squares), northern locality N1 (circles), southeastern locality SE1 (triangles), southwestern locality SW1 (diamonds); males (filled), females (empty). Note darker and more orange color in individuals from the southeastern and southwestern locality compared to those from the northern and central localities.

In March of 2020, a *Paruroctonus* observation was posted onto the citizen-science website iNaturalist by user Brian Hinds from a very small and isolated plot of relatively undeveloped land in Fresno County. In August of 2021, the authors located a cluster of additional localities for this putative species approximately 110 km to the southeast in Kern County, surrounded and fragmented by agricultural land. In April and September 2022, the authors found two additional localities approximately 50 and 65 km, respectively, southeast of the second locality cluster. In this study, we examined the alkali-sink environments associated with a large wetland system in the San Joaquin Desert, the Tulare Basin. We found the presence of a historically wide-ranging alkali-sink specialist *Paruroctonus* species and herein describe it as *P.tulare* sp. nov. (Fig. 1).

**Figure 2.**
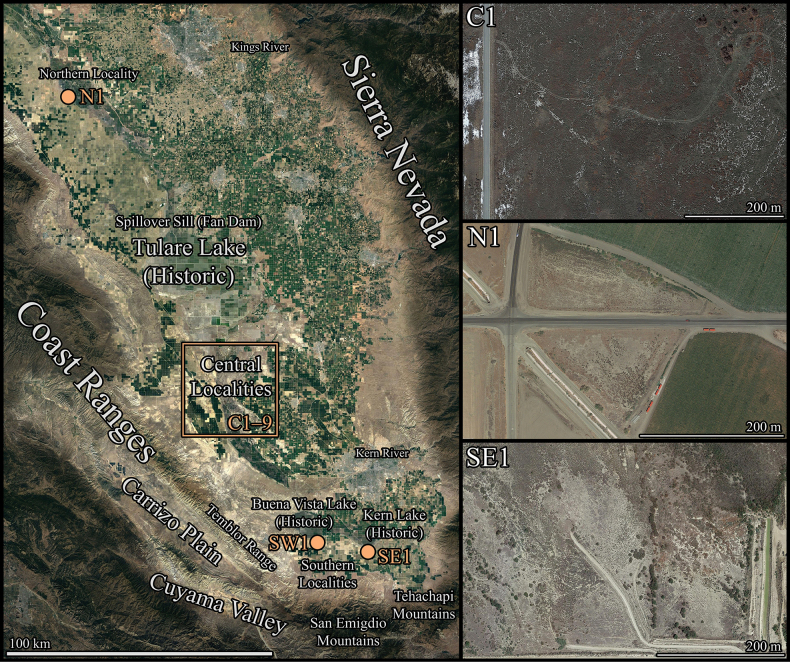
Satellite imagery of the Tulare Basin and adjacent areas indicating the geographic range of *Paruroctonustulare* sp. nov. and important geographic features within the region with locality codes indicated (left). Satellite imagery at localities C1 (February 2021), N1 (August 2018), and SE1 (April 2021).

The San Joaquin Valley (Fig. 2) is a large basin in central California, with ~ 30,000 km^2^ of lowland (< 152 m above sea level [asl]) area. It is bounded on the west and southwest by the Southern Inner Coast Ranges, on the east and northeast by the Sierra Nevada Mountains, and on the southeast by the Tehachapi Mountains (Buwalda 1954; ECORP 2007). Much of the western and southern portions of the San Joaquin Valley encompass the San Joaquin Desert, an arid region largely populated by desert-adapted species (Germano et al. 2011; Stewart et al. 2019) with the Tulare Basin (21,800 km^2^ in size, < 152 m asl) at its center. Historically, it contained the Tulare Lake, an enormous water body (1,600 km^2^) in a watershed that encompassed the Kings, Tule, Kaweah, and Kern Rivers (Atwater et al 1986; ECORP 2007).

The San Joaquin Valley is part of California’s larger Central (Great) Valley, an expansive basin between the Coast Ranges and Sierra Nevada mountains (Ikehara 1994). Formed more than 65 mya, the Central Valley remained a shallow saltwater sea until the Pliocene (Richmond et al. 2017). The water level in the Central Valley fluctuated throughout the Quaternary, with the most recent occurrence of flooding throughout the valley taking place ~ 600 kya when historic Lake Corcoran covered the entirety of the San Joaquin and Sacramento Valley floors (Atwater et al 1986; Richmond et al. 2017). Since then, the San Joaquin Valley has remained dry aside from its expansive central wetland, where standing water has persisted in approximately the same locations as the modern-day Tulare, Kern, and Buena Vista Lakes (Atwater et al 1986).

Throughout the Holocene, the level of the San Joaquin Valley’s central lakes, Tulare Lake and the smaller Buena Vista and Kern Lakes, has fluctuated heavily (Negrini et al. 2006). At the wettest times, Tulare Lake frequently spilled over the fan dam created by alluvial fans at the lake’s northern shore, and at the driest times, it occasionally dried up completely (Atwater et al. 1986). A relatively wetter period starting ~ 1,000 years before present (bp) raised the lake level, reaching a highstand of 66 m above sea level 670 years bp, and leading to several overflow events in relatively recent history (Atwater et al. 1986; Negrini et al. 2006).

Prior to European colonization, the San Joaquin Valley was largely dominated by annual wildflowers and saltbushes (*Atriplex* sp.), with significant wetland and some alkali-sink habitat near the central valley floor (Germano et al. 2011), and some areas of Valley Oak woodland and riparian habitat in the east (Kelly et al. 2005; Phillips and Cypher 2019). The historic presence of native grassland in the San Joaquin Valley has been debated, but the most recent evidence suggests that there was little to no native perennial grassland in the region (Germano et al. 2011), a finding that the poor adaptations of San Joaquin Valley fauna to grassland areas support (Germano et al. 2012). Land conversion for agriculture in the San Joaquin Valley began in the mid-1800s (Kelly et al. 2005). Valley Oak woodland areas were the first to be converted, followed shortly by wetland regions, declining to 30% and 60% of their original extents respectively by ~ 1910 (Kelly et al. 2005). Water diversion for agriculture eventually drained Tulare Lake entirely in the early to mid-1900s (Richmond et al. 2017). In their current form, wetlands and open water regions (including alkali-sinks) occupy less than 15% of their original extent (Kelly et al. 2005) while arid habitats have been reduced to ~ 35% of their original area. The lowlands (< 152 m asl) have been the most impacted with ~ 84% converted for agriculture or urbanization (Kelly et al. 2005; Dewitz and USGS 2021). Less than 5% of the land in the San Joaquin Valley remains in a natural state (USFWS 1998).

Alongside land conversion for agriculture and urbanization, a major cause of habitat degradation in the San Joaquin Valley is the spread of non-native plant species, most notably European grasses (Germano et al. 2012). While the habitat in much of the southern and western San Joaquin Valley was formerly open desert dominated by small wildflowers and sparse shrubs, it has now been largely converted to grassland by introduced grass species (Germano et al. 2012). This has caused a decline in native plant species, which are outcompeted by the larger and denser grasses, and in several native animal species, which are adapted to bare open ground and have difficulty moving through tall grass (Germano et al. 2012). Biome conversion from desert to grassland has also increased fire risk, leading to a decline in native plants that are not fire-adapted (Germano et al. 2012).

The climate in the southern San Joaquin Valley and adjacent regions most closely matches that of a desert, with hot, dry summers and mild, relatively moist winters (Germano et al. 2011). The San Joaquin Desert receives an average of 229–279 mm of rainfall yearly, although this decreases significantly to ~ 117–145 mm in the southernmost parts of the San Joaquin Valley (Germano et al. 2011). The majority of this precipitation comes in the winter months from October to March; summer months get little to no precipitation (Blunt and Negrini 2013). Evapotranspiration rates far exceed precipitation rates, so the majority of water in the San Joaquin Desert comes from the Sierra Nevada mountains, where high rainfall and runoff from snowmelt provide significant amounts of water (Blunt and Negrini 2013). Diversion of these water resources for agriculture by constructing canals and dams has heavily altered the natural state of this ecosystem by entirely eliminating seasonal flooding on the valley floor (USFWS 1998). Average summer highs in the lowland Tulare Basin reach high temperatures ~ 37–38 °C while summer lows drop to ~ 18–20 °C (Negrini et al. 2006). Current and future effects of climate change are having significant impacts on the San Joaquin Valley (Fernandez-Bou et al. 2021). These currently are and are projected to continue taking place in two major ways, increased temperatures and decreased water resources, together contributing to a more arid environment. Average precipitation in the southern San Joaquin Valley is projected to decrease by ~ 1 cm by mid-century compared to late 1900s levels (Fernandez-Bou et al. 2021). Water flow from the Sierra Nevada is also projected to decrease substantially due to climate change (Fernandez-Bou et al. 2021). During the 2011–2015 drought, the Sierra Nevada snowpack decreased by 25% due to anthropogenic causes (Berg and Hall 2017). The Sierra Nevada snowpack remains below historic levels and is projected to decrease by 20–30% compared to late 1900s levels by mid-century and by 30–64% by the end of the century, depending on the emissions scenario (Reich et al. 2018). Furthermore, the peak of water flow from the Sierra Nevada is projected to shift to 2–4 months earlier in the year by the end of the century, increasing flooding risk in winter months and substantially reducing the quantity of water available in summer months (Fernandez-Bou et al. 2021).

The San Joaquin Valley is now home to 4.3 million people, a figure that is projected to increase to 6.7 million by 2050 (Fernandez-Bou et al. 2021), potentially further reducing the extent of the remaining natural land. Temperatures in the southern San Joaquin Valley have increased by 0.6 °C during the past 70 years and are projected to continue increasing to a level 2–2.5 °C above late 20^th^ century levels by 2050, and 2.5–4.0 °C by 2100 (Fernandez-Bou et al. 2021). The frequency of extreme heat days per year in the southern valley is projected to increase from the late 20^th^ century average of 4–5 to 22–36 by 2050 and 29–58 by 2100 (Fernandez-Bou et al. 2021).

## ﻿Materials and methods

Specimens and habitats were photographed using a Canon EOS 7D camera with Canon 100 mm F/2.8, Canon 24–70 mm F/2.8, and Laowa 15 mm F/4 lenses. Stacked photographs were taken using the StackShot macro rail, combined using Helicon Focus 7, and touched up using the Gnu Image Manipulation Program and Adobe Photoshop. Scale bars on figures were constructed using pixel measurements in the photograph or illustration. Satellite imagery for maps was sourced from Google Earth and constructed using QGIS and Adobe Photoshop. Morphometric analysis plots were constructed using the Python library Matplotlib 3.5.1 (Hunter 2007).

All known records are presented in Table 1. Location codes from Table 1 (N1, C1–9, SW1, SE1) are used throughout the manuscript to refer to specific localities where *Paruroctonustulare* has been recorded.

**Table 1.** All known records of *Paruroctonustulare* sp. nov.

Locality code	Date	Locality region	Population	Locality name	Coordinates	Method	Collector/Observer
N1	28.3.2020	Northern	Northern	5.6 km SW of Tranquility	36.601727, -120.278125	Flipping	Brian Hinds
N1	18.4.2020	Northern	Northern	5.6 km SW of Tranquility	36.601727, -120.278125	Flipping	Prakrit Jain
N1	6.5.2020	Northern	Northern	5.6 km SW of Tranquility	36.601727, -120.278125	Flipping	Noah Morales
N1	20.3.2021	Northern	Northern	5.6 km SW of Tranquility	36.601727, -120.278125	Flipping	Harper Forbes, Prakrit Jain
N1	8.5.2021	Northern	Northern	5.6 km SW of Tranquility	36.601727, -120.278125	Blacklighting	Harper Forbes, Prakrit Jain
N1	15.7.2021	Northern	Northern	5.6 km SW of Tranquility	36.601727, -120.278125	Blacklighting	Harper Forbes, Prakrit Jain
C1	9.8.2021	Central	Northern	Near Kern National Wildlife Refuge 1	35.7464972, -119.5794028	Blacklighting	Prakrit Jain
C2	9.8.2021	Central	Northern	Near Kern National Wildlife Refuge 2	35.733351, -119.579182	Blacklighting	Prakrit Jain
C3	9.8.2021	Central	Northern	South of Kern National Wildlife Refuge	35.7050917, -119.5792111	Blacklighting	Prakrit Jain
C4	9.8.2021	Central	Northern	Semitropic Ridge	35.6397833, -119.5790194	Blacklighting	Prakrit Jain
C5	9.8.2021	Central	Northern	Main Drain Road	35.613811, -119.616942	Blacklighting	Prakrit Jain
C6	9.8.2021	Central	Northern	4 km E of Lost Hills	35.616969, -119.64639	Blacklighting	Prakrit Jain
C7	9.8.2021	Central	Northern	Twisselman Road	35.7316639, -119.7348389	Blacklighting	Prakrit Jain
C8	4.4.2022	Central	Northern	West Lerdo Highway	35.5001278, -119.5631722	Blacklighting	Harper Forbes, Prakrit Jain
SE1	5.4.2022	Southeastern	Southeastern	Near Kern Lake Preserve	35.0959556, -119.0490056	Blacklighting	Harper Forbes, Prakrit Jain
C9	22.3.2022	Central	Northern	Kern National Wildlife Refuge	35.733697, -119.58923	Flipping	Galen Freed-Wilhelm
SE1	17.9.2022	Southeastern	Southeastern	Near Kern Lake Preserve	35.0959556, -119.0490056	Blacklighting	Prakrit Jain
SW1	17.9.2022	Southwestern	Southwestern	South Lake Road	35.1297556, -119.2540889	Blacklighting	Prakrit Jain

Distribution modeling was conducted using Maxent 3.4.1 using default parameters (Phillips et al. 2006a). Occurrence records were sourced from iNaturalist research grade observations with location precision better than two km (and manually checked for irregularities), published papers, and personal records. A full list of records is provided in Suppl. material 1. For *Paruroctonusboreus* (Girard, 1854) and *Paruroctonussilvestrii* (Borelli, 1909), occurrence points were spatially thinned to 5 km (Kramer‐Schadt et al. 2013). All mapping calculations were conducted on a 0.01-degree grid covering the entirety of California and Nevada with the value for each variable being calculated from the center point of each grid square. Twenty-nine environmental and climatic variables were used in the species distribution modeling analysis; data sources and variable preparation procedures are described below. No transformations were applied to data unless otherwise noted. Elevation and slope degrees were sourced from LandFire 2.2.0 (LANDFIRE 2013). Bioclimatic variables 1–7 and 10–19 were sourced from WorldClim 2.1 historic 1970–2000 database at a 30 second resolution (Fick and Hijmans 2017). Snow cover days and net primary productivity were sourced from CHELSA Bioclim (Karger et al. 2017). Actual evapotranspiration, soil moisture, and vapor pressure were sourced from the terraclimate 1958–2015 data aggregates (Abatzoglou et al. 2018). Raster bands were averaged across the temporal range; the resulting data was re-scaled and smoothed to a 0.01-degree resolution using the SAGA Gaussian filter on QGIS with radius = 3 and standard deviation = 50. Soil pH, clay %, silt %, and sand % data was sourced from ISRIC soil grids at a depth of 30–60 cm to reduce the effect of recent changes (Poggio et al. 2021). Gaps were filled using the GDAL *fill nodata* function in QGIS with a 20–pixel interpolation distance and one smoothing iteration. Solar radiation was obtained from the “Global Solar Atlas 2.0, an application developed by Solargis on behalf of the World Bank Group, as Global Horizontal Irradiation from the yearly monthly totals (Global Solar Atlas 2022). Land cover was not used for the species distribution models but was used to constrain the results and was sourced from the Multi Resolution Land Characteristics Consortium National Land Cover Database (Dewitz and USGS 2021). A predicted historic species distribution was inferred by removing any outputs of the Maxent model with a cloglog value < 0.4 (indicating a < 40% probability of habitat suitability). This corresponds to the minimum training presence threshold (rounded down to the nearest 0.1). Any areas that remained disconnected and isolated were also removed. To generate maps of modern distribution, any areas with National Land Cover Database values of 11, 21–24, 81, and 82 corresponding to open water, urban development, and cultivation were removed from the historic distribution map.

Morphological analysis on various *Paruroctonus* species was done using Principal Component Analysis (PCA) and Linear Discriminant Analysis (LDA) from the Python library scikit-learn 1.0.2 (Pedregosa et al. 2011). Measurements were standardized to z scores before use in analysis. Target values for LDA were set to species separated by sex. For visualization of PCA analysis, components 2 and 3 are plotted to decrease the effect of body size (correlated strongly with the first component) on the analysis. Measurements used are provided in Table 2.

**Table 2.** Selected measurements of *Paruroctonustulare* sp. nov. specimens examined in this study. Measurements marked with an asterisk are used in morphometric analysis.

	CASENT 9101940	CASENT 9101947	CASENT 9101970	CASENT 9101967	CASENT 9101949	CASENT 9101942	CASENT 9101959	CASENT 9101954	CASENT 9101960	CASENT 9101961	CASENT 9101964	CASENT 9101946	CASENT 9101941	CASENT 9101950	CASENT 9101952	CASENT 9101944	CASENT 9101943	CASENT 9101955	CASENT 9101969	CASENT 9101962	CASENT 9101963	CASENT 9101945
Locality	C1	C2	C6	C7	C7	N1	N1	SE1	SE1	SE1	SW1	SW1	C1	C2	N1	N1	N1	SE1	SE1	SW1	SW1	SW1
Sex	male	male	male	male	male	male	male	male	male	male	male	male	female	female	female	female	female	female	female	female	female	female
Total L	46,57	42,04	41,45	44,36	53,24	51,96	49,03	51,14	46,97	43,02	46,75	48,87	50,01	50,52	48,09	54	47,07	44,1	46,89	44,82	45,21	45,97
Prosoma L*	5,82	5,64	5,12	5,87	6,62	6,29	6,22	5,99	5,88	5,51	5,85	5,7	6,74	6,96	6,63	7,4	6,48	6,1	6,12	5,85	6,17	6,18
Prosoma posterior W	5,53	5,24	4,99	5,74	5,74	5,72	5,9	5,44	5,54	5,33	5,54	5,43	6,74	6,99	6,84	7,05	6,36	5,76	5,95	5,36	6,08	5,87
Prosoma interocular W	4,26	4,08	4,01	4,7	4,47	4,68	4,71	4,42	4,37	4,16	4,27	4,43	5,16	5,4	4,81	5,87	4,92	4,35	4,46	4,43	4,95	4,71
Mesosoma L	11,81	10,07	9,15	10,2	13,89	14,3	11,49	13,86	12,36	10,5	12,14	14,43	13,62	13,22	12,36	14,65	13,43	11,59	14,59	12,78	10,75	12,43
Metasoma L	29,61	26,63	25,62	29,92	33,2	31,01	30,89	30,71	28,75	27,23	28,58	29,53	31,3	31,42	29,59	32,86	27,42	26,98	27,01	26,59	27,66	26,7
Metasoma I L*	3,4	3,03	3,02	3,57	3,68	3,58	3,62	3,57	3,29	3,18	3,32	3,27	3,58	3,42	3,26	3,84	3,11	3,05	3,15	3,07	3,25	3,04
Metasoma I W*	3	2,9	2,6	3,23	3,44	3,36	3,44	3,24	3,19	2,95	3,18	3,07	3,53	3,56	3,75	3,58	3,5	3,13	3,11	3,12	3,14	3,11
Metasoma I H*	2,38	2,25	2,06	2,49	2,81	2,63	2,66	2,56	2,52	2,36	2,57	2,54	2,8	2,66	2,77	3,05	2,75	2,32	2,35	2,42	2,24	2,45
Metasoma II L*	3,95	3,59	3,49	4,12	4,3	4,31	4,02	4,06	3,82	3,71	3,9	3,97	4,05	3,88	3,69	4,17	3,53	3,5	3,53	3,43	3,62	3,51
Metasoma II W*	3	2,9	2,56	3,13	3,5	3,25	3,3	3,25	3,15	3	3,15	3,14	3,47	3,57	3,56	3,77	3,4	3,1	3,11	3	3,2	3,08
Metasoma II H*	2,39	2,25	2,13	2,54	2,8	2,65	2,64	2,54	2,48	2,38	2,53	2,48	2,82	2,84	2,84	3,04	2,67	2,38	2,34	2,38	2,56	2,48
Metasoma III L*	4,47	3,82	3,81	4,21	4,64	4,53	4,39	4,28	4,1	4,04	4,19	4,28	4,35	4,22	3,99	4,51	3,87	3,68	3,79	3,54	3,82	3,84
Metasoma III W*	2,91	2,76	2,59	2,97	3,35	3,19	3,22	3,1	3,05	2,88	3,01	2,96	3,3	3,46	3,43	3,58	3,21	2,95	2,98	2,98	3,01	2,96
Metasoma III H*	2,46	2,25	2,11	2,55	2,83	2,74	2,66	2,58	2,53	2,4	2,54	2,57	2,87	2,83	2,83	3,07	2,67	2,4	2,4	2,4	2,59	2,54
Metasoma IV L*	5,24	4,59	4,33	5,08	5,57	5,21	5,22	5	5	4,64	4,9	4,89	5,14	5,04	4,7	5,44	4,64	4,35	4,44	4,04	4,54	4,42
Metasoma IV W*	2,63	2,54	2,27	2,82	3,22	2,9	2,95	2,83	2,89	2,68	2,77	2,77	3,13	3,15	2,96	3,58	2,97	2,73	2,75	2,75	2,66	2,78
Metasoma IV H*	2,44	2,25	2,15	2,53	2,8	2,67	2,68	2,55	2,54	2,42	2,53	2,51	2,91	2,87	2,84	3,03	2,67	2,43	2,34	2,43	2,43	2,55
Metasoma V L*	7,02	6,44	5,94	7,12	8,01	7,28	7,44	7,02	6,92	6,51	6,7	6,76	7,43	7,29	6,99	7,69	6,75	6,2	6,32	6,04	6,36	6,38
Metasoma V W*	2,57	2,35	2,2	2,6	2,97	2,78	2,72	2,72	2,68	2,51	2,69	2,7	2,99	2,93	2,99	3,23	2,84	2,62	2,68	2,56	2,68	2,63
Metasoma V H*	2,3	2,12	2,03	2,35	2,71	2,59	2,42	2,34	2,54	2,15	2,44	2,26	2,72	2,67	2,76	2,89	2,52	2,33	2,27	2,27	2,35	2,37
Telson L*	6,81	6,14	5,81	6,56	7,43	6,61	6,87	6,97	6,78	6,46	6,67	6,51	7,38	7,91	6,88	7,75	6,4	6,51	6,7	6,31	6,81	6,55
Vesicle L*	4,96	4,78	4,43	4,96	5,73	4,68	5,54	5,34	5,34	4,88	5,2	4,9	5,56	6,02	4,86	5,24	5,11	4,88	4,88	4,62	4,46	4,48
Vesicle W*	2,3	2,04	1,99	2,51	2,64	2,41	2,7	2,36	2,47	1,94	2,49	2,36	2,74	2,82	2,71	3,09	2,48	2,57	2,58	2,4	2,39	2,41
Vesicle H*	1,76	1,61	1,54	1,91	2,04	1,84	1,97	1,82	1,94	1,62	1,86	1,69	2,17	2,24	2,25	2,3	1,93	1,8	1,91	1,72	1,87	1,88
Aculeus L	1,92	1,86	1,46	2,14	1,53	2,33	1,43	1,48	1,78	1,72	1,64	1,38	2,51	2,56	2,2	2,79	2,23	1,37	1,53	1,55	2,11	1,49
Pedipalp L	20,8	19,24	17,64	20,98	22,77	21,85	21,08	19,72	19,29	18,77	20,04	18,84	23,36	23,2	21,18	24,15	20,77	19,08	19,72	17,67	19,8	19,12
Pedipalp femur L*	4,94	4,34	4,29	4,86	5,47	4,95	5,06	4,97	5,04	4,5	4,91	4,77	5,45	5,5	5,13	5,43	4,75	4,63	4,73	4,32	4,69	4,47
Pedipalp femur W*	1,6	1,49	1,52	1,64	1,92	1,56	1,75	1,75	1,76	1,62	1,75	1,79	1,83	1,93	1,74	1,78	1,79	1,74	1,65	1,69	1,58	1,52
Pedipalp femur H*	1,43	1,34	1,2	1,58	1,53	1,48	1,4	1,37	1,31	1,27	1,4	1,39	1,69	1,56	1,57	1,84	1,64	1,34	1,41	1,35	1,34	1,42
Pedipalp patella L*	5	5,55	4,48	4,95	5,65	5,15	5,19	4,85	4,73	4,68	5,1	4,92	5,81	5,63	5,61	5,91	5,33	4,86	5,17	4,95	5,03	4,95
Pedipalp patella W*	2,09	1,96	1,77	2,03	2,3	2,16	2,09	2,04	2,09	1,93	2,05	1,98	2,35	2,32	2,23	2,55	2,31	2,1	2,1	2,03	1,99	2,08
Pedipalp patella H*	1,93	1,78	1,69	2,01	2,2	2,1	1,97	1,99	2,08	1,94	2,09	2,02	2,26	2,27	2,06	2,38	1,97	2,01	2,04	1,9	2,03	2
Chela L*	9,81	9,11	8,56	9,67	10,92	9,65	9,94	9,64	9,72	9,13	9,85	9,48	10,88	11,23	9,88	11,02	9,74	9,44	9,79	9,09	9,68	9,71
Manus W*	3,85	3,76	3,36	4,13	4,68	4,26	3,98	4,44	4,44	4,19	4,61	4,25	4,19	4,09	3,75	4,63	3,76	4,08	3,98	3,8	4	3,94
Manus T*	2,79	2,6	2,5	2,9	3,44	3,08	3,04	3,22	3	2,88	3,25	2,97	3,21	3,11	2,79	3,39	2,82	2,9	2,94	2,82	2,9	2,74
Finger fixed L	4,58	3,86	3,55	4,57	4,35	4,63	3,81	3,54	3,65	3,59	3,79	3,75	5,31	5,18	4,59	4,88	4,33	3,61	4,32	3,88	4,16	4,2
Finger movable L*	6,22	6,05	5,56	6,18	6,62	5,96	6,29	6,19	6,34	5,81	6,52	6,27	6,86	7,04	5,85	6,58	6,02	5,98	6,15	5,96	6,39	6,18
Pectine L*	5,64	6,12	4,86	5,95	7,02	6,88	6,56	6,06	6,2	5,28	5,81	5,92	5,03	4,93	4,78	5,31	4,47	3,76	4,24	4,13	4,5	4,33
Pectine W	1,53	1,69	1,26	1,86	1,82	1,98	1,4	1,66	1,32	1,55	1,56	1,41	1,53	1,04	1,04	1,92	1,01	0,98	1,05	0,97	1,19	1,13

DNA was extracted from eight specimens using the QIAamp Micro DNA extraction kit, following the tissue extraction protocol but tripling the amount of proteinase K (from 20 μl to 60 μl) and with the optional step of including carrier RNA. The gene cytochrome oxidase subunit I (COI) was amplified via polymerase chain reaction (PCR) for sequencing using the primers HCO2198/LCO1490 (Folmer et al. 1994). PCR protocol was as follows: a) 94 °C for two minutes, b) 95 °C for 30s, c) 48 °C for 30s, d) 72 °C for 95s, e) 72 °C for seven minutes, and f) hold at 4 °C, repeating steps b) through d) for 40 cycles. PCR products were analyzed for a gel electrophoresis band using ethidium bromide and a PCR cleanup step was implemented with ExoSAP-IT. Sanger sequencing was conducted externally through ELIM BIOPHARM (Hayward, California, USA). ABI files were then assembled de novo in Geneious Prime 2022 (Geneious Prime 2023) and aligned using the program MAFFT (Katoh and Standley 2013). Sequences were manually examined for gaps or stop codons in AliView and then exported to PHYLIP format for use in the program PopART (Larsson 2014; Leigh and Bryant 2015). In PopART, a haplotype network was constructed using the integer neighbor-joining (IntNJ) method described in Leigh and Bryant (2015). Sequences used in the haplotype network were submitted to GenBank and accession numbers are available in Suppl. material 2.

Nomenclature and measurements largely follow Stahnke (1970) with a few exceptions. Basitarsal and telotarsal spine and setal nomenclature follows McWest (2009). Metasomal carinal nomenclature follows González-Santillán and Prendini (2013) with “lateral median” replaced with “lateral supramedian,” consistent with Sissom and Francke (1981). Metasomal setae follow the name of their associated carinae. Pedipalp carinae and trichobothrial nomenclature follows González-Santillán and Prendini (2013). Setae on the manus follow the name of their associated carinae.

All elements in the diagnosis, unless otherwise noted, are not sexually dimorphic and apply to late instar juveniles as well. Counts and measurements separated by a “/” indicate a difference on the left/right sides of a single specimen, while those separated by a “–” indicate a range across multiple examined specimens. Setal counts used in the diagnosis are taken as the maximum number of macrosetae on either the right or left side of the individual scorpion (e.g., if a scorpion had two macrosetae on the left and three on the right, the setal count would be “3”); the statistic “Max Range” in Suppl. material 3 is reported in the same way. The “Range” statistic in Suppl. material 3 is the minimum macrosetae (on either side) observed to the maximum observed; left and right sides are averaged for the “Average” and “Variance” statistics. Total length includes telson and is not calculated additively. Measurements were made using digital calipers and are given in mm. Specimens examined and photographed are preserved in 95% ethanol. Preserved specimens examined are deposited at the California Academy of Sciences (**CAS**).

## ﻿Results

### ﻿Haplotype network

(Fig. 3)

**Figure 3.**
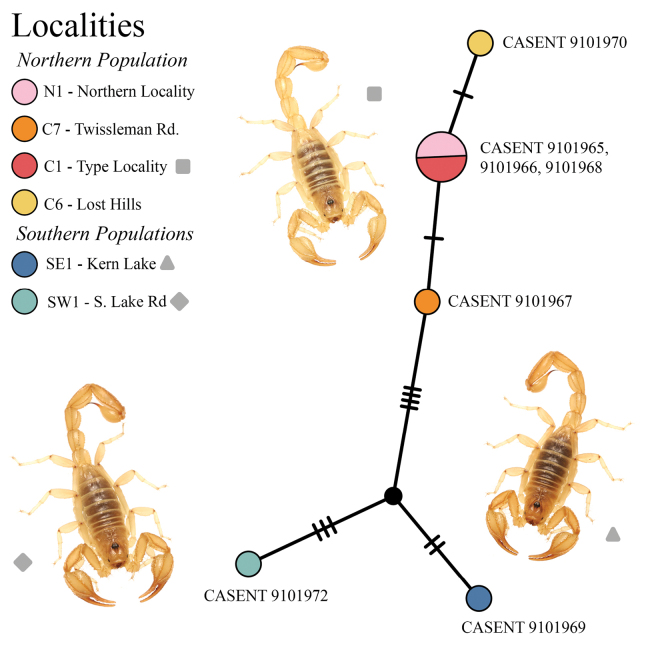
A haplotype network including a number of individuals of *Paruroctonustulare* sp. nov. from across their geographic range, constructed using the neighbor-joining method integer (IntNJ) in PopART. Pictured: central locality (square) southeastern locality SE1, (triangle), southwestern locality SW1, (diamond). Colored circles correspond to localities indicated in the legend, hash marks indicate individual base pair differences between each sample, size of the circle corresponds to the number of samples belonging to that haplotype, and solid black circle corresponds to a hypothesized ancestor not represented by the data.

The integer neighbor-joining haplotype network demonstrated population structure, albeit limited, with homogeneity between central localities C1, C6, and C7 (Kern National Wildlife Refuge) and the northern locality N1 (San Joaquin/ Tranquility), but otherwise isolated haplotypes amongst all other localities (Figs 2, 3). We identified three putative populations, each separated by fewer than four single nucleotide polymorphisms: a northern population constituting the northern (N1) and central (C1–9) cluster localities, a southeastern population (SE1), and a southwestern population (SW1). While individuals from the southeastern and southwestern localities share morphological similarities, their genetic distance from each other and from the northern population are approximately equivalent.

### ﻿Morphometric analysis

(Fig. 4)

PCA clustered all *P.tulare* sp. nov. points into minimum convex polygons distinctly separate in morphospace from all other species aside from *P.soda*. Overall, intraspecific overlap among the three populations was higher in males than in females. For principal component 2, pectine length (r = 0.93), and metasomal segment length (0.30 < r < 0.47), were the most important factors. Based on principal component 2, males of different species were more distinct in morphospace than females. For principal component 3, vesicle width (r = 0.39), vesicle height (r = 0.31), metasoma V length (r = 0.23), and telson length (r = 0.23), were the most important factors. Factor 3 appeared to be the most diagnostic for *P.tulare* sp. nov. within the morphospace. LDA resulted in distinct polygons for each species with no overlap. Following a similar pattern as the PCA, the first discriminant function was dominated by pectine length (r = 0.61), and the majority of the second discriminant function was telson morphology (vesicle height, r = 0.32; vesicle width, r = 0.24). This discriminant was the most diagnostically informative for *P.tulare* sp. nov. All populations of *P.tulare* sp. nov. overlap significantly in morphospace on the LDA plot but there is some minor differentiation in the PCA plot.

**Figure 4.**
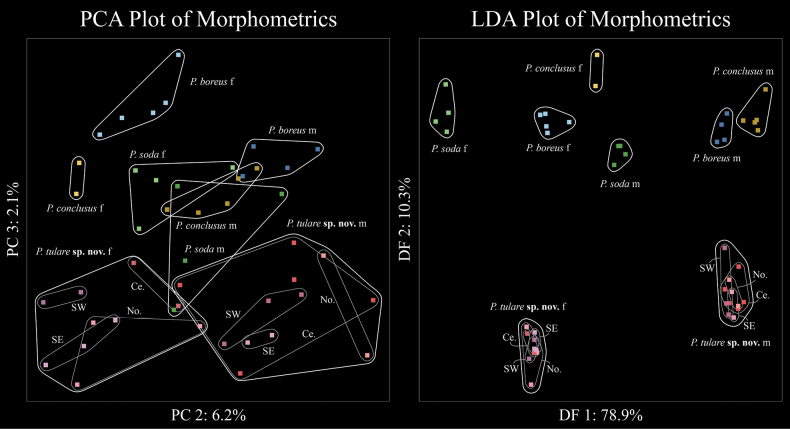
Principal component analysis plot of *Paruroctonustulare* sp. nov. and other morphologically or ecologically similar species using standardized morphometrics (left) with components 2 and 3 pictured to reduce the effect of body size. Linear discriminant analysis plot of *Paruroctonustulare* sp. nov. and other morphologically or ecologically similar species using standardized morphometrics and based on species identifications (right) with factors one and two pictured.

### ﻿Distribution model

(Fig. 5)

The habitat model generated using Maxent returned a high AUC value of 0.999, indicating high predictive differentiation between presence points and background regions, although this value is potentially inflated by the large number of unsuitable background points included within the analysis grid (Merow et al. 2013). The highly altered state of the San Joaquin valley limits sampling throughout much of the projected historic distribution of *P.tulare* sp. nov., leading to increased sampling bias and creating limitations to interpreting the habitat model and using AUC as a measure of its performance (Merow et al. 2013).

**Figure 5.**
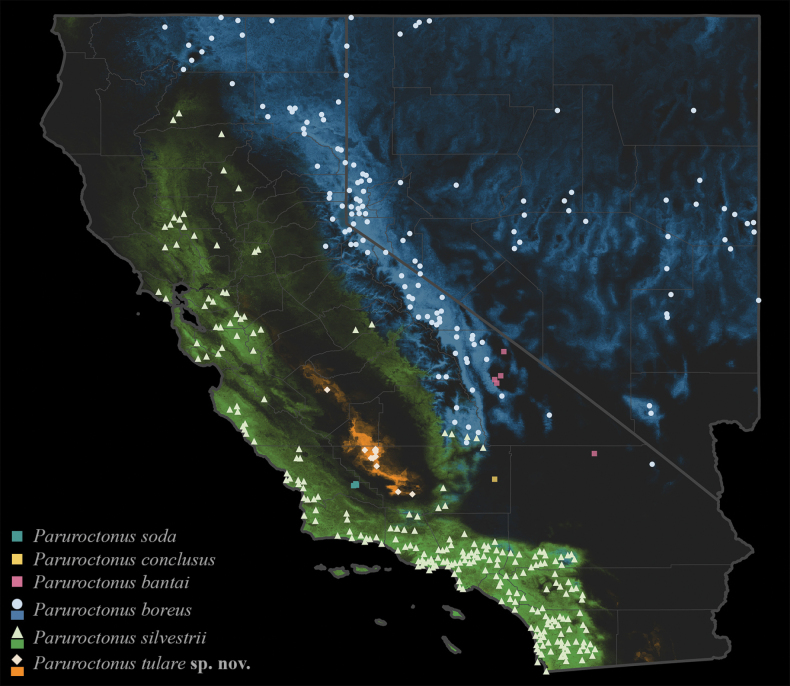
Maximum entropy niche models of *Paruroctonustulare* sp. nov. and other selected *Paruroctonus* species indicating ecological uniqueness based on 29 environmental variables. The distribution models depicted in colored shading represent: *Paruroctonusboreus* in blue, *Paruroctonussilvestrii* in green, and *Paruroctonustulare* sp. nov. in orange.

Precipitation of the warmest quarter, vapor pressure, percent sand in soil, topographic slope, and net primary productivity were found to be the most important variables in the model. Variables with a high positive correlation with the modeled habitat suitability for *P.tulare* sp. nov. include silt and clay percentage in the soil; pH; solar radiation; various measures of temperature highs; and temperature range. The minimum cloglog output value at any training presence point was 0.442 at N1 and the 10^th^ percentile training presence returned a cloglog output of 0.504. The predicted historic species distribution covered 2194 km^2^ with 1858 km^2^ of this area excluded due to flooding, agriculture, or urban development in modern times (Fig. 5).

### ﻿Systematics

#### ﻿Family Vaejovidae Thorell, 1876

**Genus *Paruroctonus* Werner, 1934**

##### ﻿ Paruroctonus tulare sp. nov.

Taxon classificationAnimaliaScorpionesVaejovidae

1F51123D-CBF6-5CC7-A554-64F135E064F8

https://zoobank.org/2E6A610D-20AA-4086-96BA-CF8F6EBA35EC

Figs 1 , 2 , 3 , 4 , 5 , 6 , 7 , 8 , 9 , 10 , 11 , 12 , 13 , 14 , 15 , 16 , 17 , 18 , Tables 1 , 2 , Suppl. material 3

###### Type material.

***Holotype*.** USA • 1 ♂; California, Kern County, near Kern National Wildlife Refuge; 35.7465, -119.5794; 67 m a.s.l.; 8 August 2021; collector leg Prakrit Jain; collected at night using handheld UV light; CASENT 9101940.

***Paratypes*.** USA • 3 ♀; same data as holotype; CASENT 9101941, 9101968 • 1 ♂, 1 ♀; California, Kern County, near Kern National Wildlife Refuge; 35.7334,-119.5792; 68 m a.s.l.; 8 August 2021; collector leg Prakrit Jain; collected at night using handheld UV light; CASENT 9101947, 9101950 • 2 ♂; California, Kern County, near Twisselman Road; 35.7317,-119.7348; 68 m a.s.l.; 8 August 2021; collector leg Prakrit Jain; collected at night using handheld UV light; CASENT 9101967, 9101949 • 1 ♂; California, Kern County, 4 km E of Lost Hills; 35.6170,-119.6464; 72 m a.s.l.; 8 August 2021; collector leg Prakrit Jain; collected at night using handheld UV light; CASENT 9101970.

###### Additional material examined.

USA • 1 ♀; California, Fresno County, 5.6 km SW of Tranquility; 36.6017, -120.2781; 52 m a.s.l.; 18 April 2020; collector leg Prakrit Jain; found in the day under discarded concrete; CASENT 9101943 • 1 ♀; California, Fresno County; same locality and coordinates; 6 May 2020; collector leg Noah Morales; found in the day under discarded concrete; CASENT 9101944 • 1 ♀; California, Fresno County; same locality; 8 May 2021; collector leg Harper Forbes, Prakrit Jain; collected at night using handheld UV light; CASENT 9101952 • 3 ♂, 2 juv. ♀; California, Fresno County; same locality; 15 July 2020; collector leg Harper Forbes, Prakrit Jain; collected at night using handheld UV light; CASENT 9101942, 9101965, 9101966, 9101953, 9101959 • 1 ♂, 2 ♀, 2 juv. ♀; California, Kern County, near Kern Lake Preserve; 35.0960, -119.0490; 100 m a.s.l.; 5 April 2022; collector leg Harper Forbes, Prakrit Jain; collected at night using handheld UV light; CASENT 9101954, 9101955, 9101969, 9101957 • 2 ♂; same locality; 17 September 2022; collector leg Prakrit Jain; collected at night using handheld UV light; CASENT 9101960, 9101961 • 2 ♂, 3 ♀; California, Kern County, S Lake Road; 35.1298, -119.2541; 99 m a.s.l.; 17 September 2022; collector leg Prakrit Jain; collected at night using handheld UV light; CASENT 9101962, 9101963, 9101964, 9101945, 9101946.

###### Diagnosis.

Four additional species of *Paruroctonus* are known from the San Joaquin Desert and its surrounding ranges (Sierra Nevada, Southern Inner Coast Ranges, Tehachapi Mountains): *P.boreus*, *P.silvestrii*, *P.variabilis*, and *P.soda*. Comparisons are provided against these four as well as an alkali-sink scorpion species from the northern Mojave Desert, *P.conclusus*. *Paruroctonustulare* sp. nov. is endemic to alkali-sink environments associated with wetlands along the center of the San Joaquin Valley, where it is allopatric with all *Paruroctonus* except *P.variabilis*. *Paruroctonustulare* sp. nov. differs from the other aforementioned *Paruroctonus* species by a combination characteristics categorized below.

###### Comparisons.

*Paruroctonustulare* sp. nov. can be differentiated from *P.variabilis* and *P.silvestrii* using the following quantitative morphological (A), qualitative morphological (B), and morphometric (C) characteristics:

A) Ventrolateral and ventral submedian setae on metasomal segments I–IV, excluding any on the posterior margin of their respective segments, follow the patterns 1–2; 2, 3, 3, 3; and 2, 2–3, 2–3, 3; in *P.variabilis* 3, 3–5, 4–5, 5–6 and 3–4, 4, 4–5, 4–8; and in *P.silvestrii* 2, 3, 3, 3–4 and 2–3, 3, 3–4, 3–4. Dorsal median and retrolateral median carinae on the manus have no more than 1 large macroseta each, *P.variabilis* has 2–4 and 3–5 respectively, and *P.silvestrii* has 1–2 and 2–3 respectively. Pedipalp patella has 0–1 (typically 1) large retrolateral medial macroseta, in *P.variabilis* 3–5, and in *P.silvestrii* 2–4.

B) Chelal fingers of adult males with moderate to heavy scalloping leaving a large proximal gap when closed, absent in *P.variabilis* and *P.silvestrii*. Dark fuscous patterning sometimes present on the pedipalps and on the carapace outside the interocular area in *P.variabilis* and *P.silvestrii*, absent in *P.tulare* sp. nov.

C) The length:width ratio of metasomal segment V of adult males (females) is 2.49–2.74 (2.34–2.49), in *P.variabilis* 2.85–3.02 (2.63–2.89), in *P.silvestrii* 2.72–3.01 (2.46–2.63). Chela length:manus width, chela length:manus thickness ratios of adult males (females) are 2.14–2.55, 2.99–3.52 (2.31–2.75, 3.22–3.61), in *P.variabilis* 2.49–3.10, 3.39–4.03 (2.90–3.37, 3.94–4.27), in *P.silvestrii* 2.59–2.70, 3.36–3.65 (2.69–3.06, 3.58–4.15).

*Paruroctonustulare* sp. nov. can be differentiated from *P.boreus* using the following characteristics:

B) Dark fuscous patterning on the carapace outside the interocular area is absent or pale, present in *P.boreus*. Ventral retrolateral and retrolateral median carinae on the pedipalp patella sparse and smooth, dense and thick in *P.boreus*.

C) The length:width ratio of metasomal segments V in adult males (females) is 2.49–2.74 (2.34–2.49), in *P.boreus* 3.00–3.05 (2.57–2.79). The length:height ratio of metasomal segment V in adult males (females) is 2.72–3.07 (2.53–2.78), in *P.boreus* 3.25–3.63 (2.90–3.22).

*Paruroctonustulare* sp. nov. can be differentiated from *P.soda* using the following characteristics:

A) Dorsolateral, ventrolateral, and ventral submedian setae on metasomal segments I-V, excluding any on the posterior margin of their respective segments, follow the patterns 0,0–1,1,1–2; 2,3,3,3; and 2,2–3,2–3,3; and in *P.soda* are 0,0,0,1; 1–2,2,2,2–3; and 1–2,2,2,2 respectively. Prolateral ventral & prolateral median carinae on the manus have 1–2 (typically 2) and 2 macrosetae each, no more than 1 in *P.soda*. Pedipalp patella has 0–1 (typically 1) large retrolateral medial macroseta, absent in *P.soda*.

C) Length:width of metasomal segments III, IV in adult males (females) is 1.34–1.54, 1.73–1.99 (1.16–1.32, 1.47–1.71), in *P.soda* 1.25–1.30, 1.56–1.70 (1.11–1.23, 1.40–1.63). Length:height of metasomal segments III, IV in adult males (females) is 1.62–1.82, 1.92–2.15 (1.41–1.58, 1.58–1.90), in *P.soda* 1.50–1.61, 1.76–1.97 (1.46–1.56, 1.60–1.71).

*Paruroctonustulare* sp. nov. can be best differentiated from *P.conclusus* using the following characteristics:

B) Dorsal median carina does not noticeably curve prolaterally between the *db* and *dsb* trichobothria, curves in *P.conclusus*.

C) Length:width ratio of metasomal segment V in adult males is 2.49–2.74, in *P.conclusus* 2.86–3.05. Length:height ratio of metasomal segment V in adult males is 2.72–3.07, in *P.conclusus* 3.10–3.52.

###### Holotype description.

**Figure 6.**
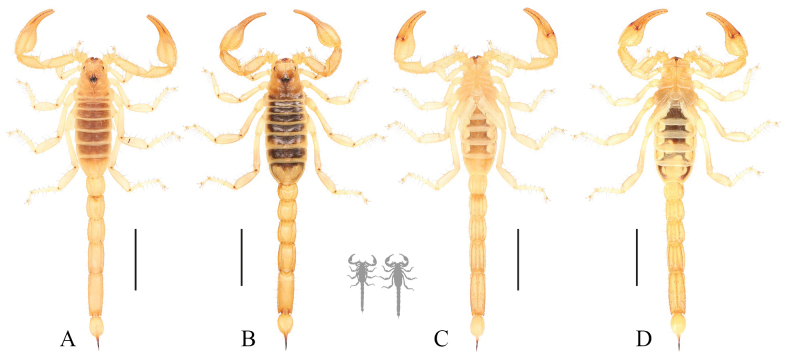
Habitus photos of *Paruroctonustulare* sp. nov. from the type locality **A, B** dorsal and **C, D** ventral **A, C** holotype male and **B, D** paratype female. Scale bars: 10 mm, silhouettes to scale.

**Figure 7.**
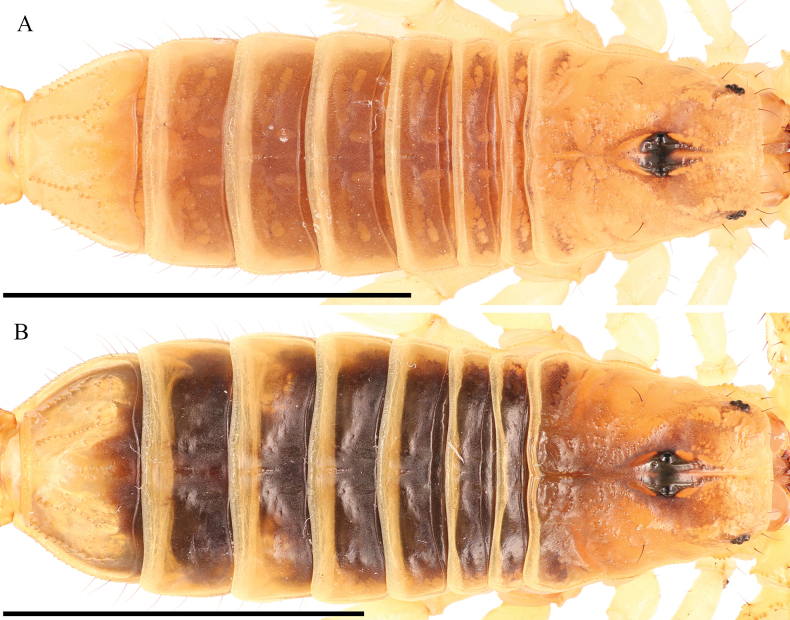
Dorsal trunk of *Paruroctonustulare* sp. nov. from the type locality **A** holotype male **B** paratype female. Scale bars: 10 mm.

***Coloration*** (Figs 1, 6, 7). Carapace tan. Scattered fuscous markings present throughout, fuscousity especially prominent along the posterior and lateral edges of the interocular triangle, at the posterior-lateral corners of the carapace, and directly posterior to the ocular tubercle. Tergites I–VI darker brown with lighter, yellowish posterior and lateral margins. Fuscousity does not extend to the posterior or lateral margins of tergites I–VI. Tergite VII with faint fuscousity concentrated near the anterior margin. Legs pale yellow with some faint fuscousity near the joint of the femur and patella. Pedipalps tan-orange with darker carinae and orange fingers. Metasoma tan, with slightly darker carinae. Weak metasomal fuscousity is present on the ventral submedian and ventrolateral carinae on segments II-IV and on the ventral surface of segment V, with the intensity increasing on subsequently posterior segments. Telson pale yellow, base of aculeus dark reddish-brown, and aculeus black. Sternites III–VI dark brown with brown spiracles; tergite VII tan. Pectines, sternum, and genital operculum tan-cream.

**Figure 8.**
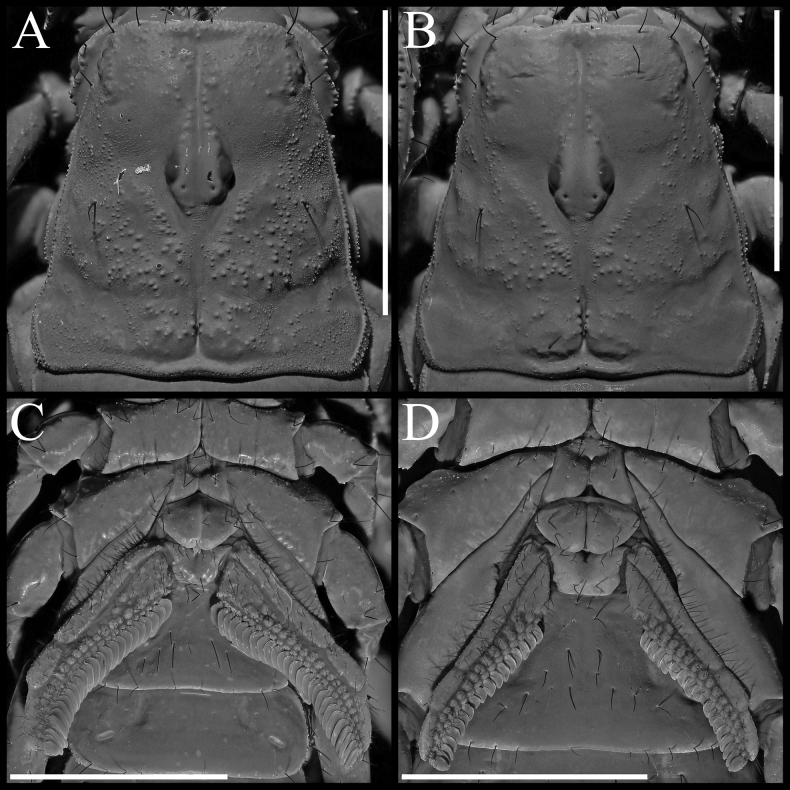
*Paruroctonustulare* sp. nov. from the type locality **A, B** carapace and **C, D** sternopectinal region **A, C** holotype male and **B, D** paratype female. Shown under ultraviolet illumination. Scale bars: 5 mm.

***Carapace*** (Figs 7, 8). Anterior margin approximately straight to slightly convex with three pairs of distinct macrosetae. Posterior median portion of the carapace sparsely and irregularly granular, large granules decrease in size and density anteriorly and laterally. Anterior and median regions also have even and very fine granules. Posterior, lateral, and anterior margins finely crenulate. Posterior median sulcus narrow and moderately deep, mostly smooth. Anterior median, median ocular, and posterior marginal sulci shallow, free of large granules. Lateral ocular and posterior lateral sulci broad and shallow, free of large granules. Interocular region of the carapace smooth with sparse granules anteriorly. Median ocelli separated by a distance greater than the width of one oculus. Three lateral ocelli present on each side. Single pairs of macrosetae present posterior to the median ocelli, situated between the lateral ocelli and the margin of the carapace, and approximately halfway between the posterior median sulcus and the posterior margin of the carapace, in line with the posterior edge of the ocular tubercle.

***Mesosoma*** (Fig. 7). Tergites I–VI smooth except on the posterior-lateral fourth on each side, which is weakly granular to granular, with the intensity of granulation increasing on subsequent segments, and the posterior margin, which is smooth to weakly granular, once again with the intensity of granulation increasing on subsequent segments. Median longitudinal carina weak, smooth on I and weakly irregularly crenulate on II–VI. Submedian longitudinal sulci indistinct. One pair of small posterior submedian setae on tergites I–V. Tergite VII smooth dorsally; dorsolateral and lateral areas sparsely granular. Lateral marginal carina finely crenulate; dorsolateral and dorsal submedian carinae strongly crenulate. Median longitudinal carina weakly crenulate. Sternites III–VI sparsely setose and smooth. Sternite VII sparsely and very weakly granular medially and finely granular laterally, with ventral submedian carinae weakly crenulate and lateral marginal carinae finely crenulate.

***Pectines*** (Fig. 8). Elongate and densely hirsute, with 26/25 tightly packed teeth. Middle lamellae circular distally, highly irregular in size and shape proximally; approximately 21/19 distinct and separated sclerotized sections are visible under ultraviolet illumination.

***Genital operculum*** (Fig. 8). Sclerites triangular with rounded corners, approximately as wide as long. Overlapping medially and separated slightly only at the posterior edge, with protruding genital papillae. Several macrosetae present on each sclerite.

***Sternum*** (Fig. 8). Type 2 (sensu Soleglad and Fet 2003) with posterior emargination absent, apex deep, slightly wider than long, smooth except very finely granular along the slopes of the apex. Three pairs of macrosetae.

**Figure 9.**
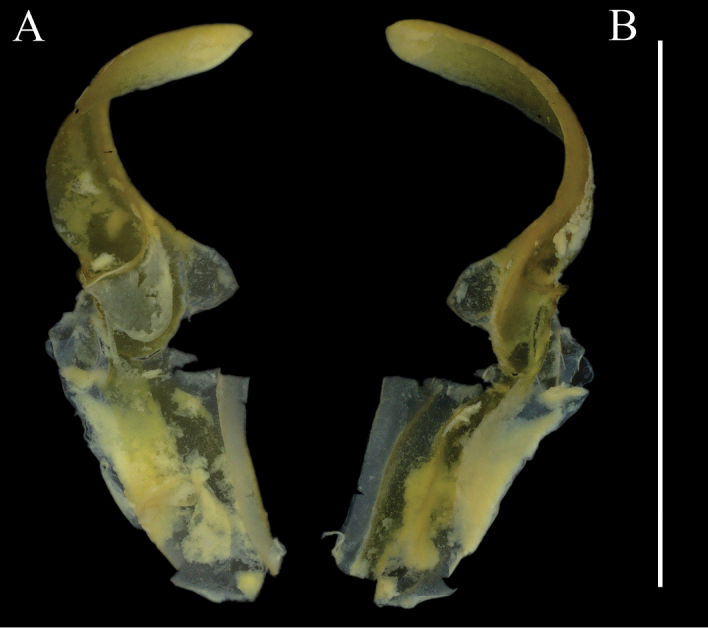
Hemispermatophore of paratype *Paruroctonustulare* sp. nov. adult male **A** ectal and **B** ental view. Scale bar: 5 mm.

***Hemispermatophore*** (Fig. 9). Hemispermatophore approximately equal in width from pedicel to stalk, three-fold bauplan (Monod et al. 2017). Stalk wide, relatively straight, and dorso-ventrally flattened. Distal carina and lamellar hook sclerotized, lamellar hook bifurcate at terminus. Mating plug weakly sclerotized, moderate in size with a wide bilobed base and relatively long stem terminating in a barb.

***Legs*** (Fig. 6). Carinae: Retroventral carina on femur finely crenulate. Superior carinae on Leg I femur weakly and finely crenulate, decreasingly distinct on subsequent legs. Proventral carina sparsely, finely and weakly crenulate on Leg I patella, decreasingly distinct on subsequent legs and nearly absent by leg IV. Intercarinal spaces on legs smooth with sparse, fine granules on the femur.

Telotarsi: Telotarsal retroinferior terminal macrosetae on legs I–IV 1, 2, 2, 2; other telotarsal retroinferior macrosetae on the distal half of telotarsi I–IV 1, 1, 2, 2. Two telotarsal retromedial macrosetae on each leg, with one always at the retromedial terminal position. Two large telotarsal retrosuperior macrosetae on each leg, an additional smaller distal one on each leg except on R leg I. Single proinferior terminal macroseta on each leg. Single proinferior distal macroseta on each leg with an additional one on L leg I, one medial proinferior macroseta on legs II–IV. Two telotarsal promedial macrosetae on legs I–III at terminal and distal positions; one on leg IV in terminal position. Two large telotarsal prosuperior macrosetae on each leg in terminal and medial positions. Telotarsal superior macroseta present on all legs; telotarsal superioterminal macroseta present on all legs.

Basitarsi: Three basitarsal spine rows present on legs I–II; proventral and retroventral spine rows equally dense and retrosuperior spine row less dense. The retroventral spine row extends approximately four-fifths the entire length of the segment, the proventral spine row extends through approximately half the segment, and the retrosuperior spine row extends irregularly through approximately half. On leg III, the proventral spine row is absent and the other two are heavily reduced in density. On leg IV, both the proventral and retroventral spine rows are absent and the retrosuperior spine row is heavily reduced in density, almost absent. Basitarsal retroventral macrosetae, excluding only the distal retroventral spinoid macroseta, on legs I–IV follow the pattern 3/3, 6/6, 6/6, 5/6, with variably sized setae. Spinoid basitarsal proventral macrosetal pattern on legs I–IV is 2/2, 2/3, 3/3, 3/3; a thinner terminal ventral macroseta is present on legs II–IV. Superior basitarsal macrosetae on legs I–IV consist of two spinoid macrosetae at the distal and mid-retrosuperior positions, as well as a small retrosuperior macroseta on L leg III; one prosuperior macrosetae at the distal position along with other prosuperior macrosetae occasionally present at variable positions; one macroseta at the distal superiomedian position adjacent to the distal retrosuperior spinoid macroseta, absent on L leg IV. Superiomedian macrosetae on legs I-IV follow the pattern 6/5, 6/6, 6/6, 4/5. Prolateral macrosetae on legs I–IV, excluding one on the distal margin, follow the pattern 3/3, 3/3, 3/3, 2/2.

**Figure 10.**
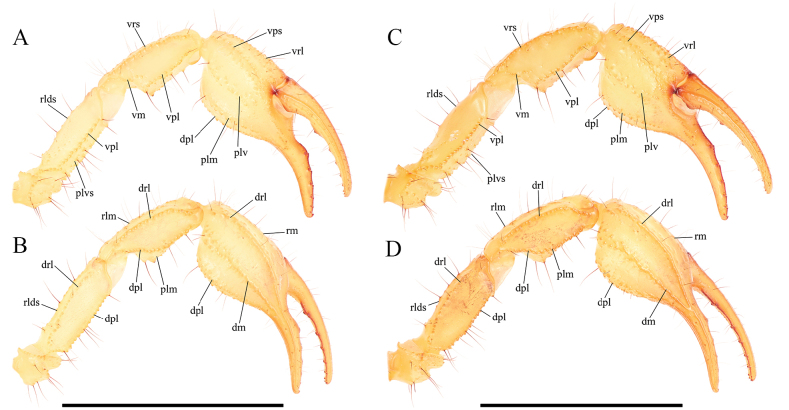
Pedipalp of *Paruroctonustulare* sp. nov. from the type locality **A, B** holotype male and **C, D** paratype female **A, C** ventral aspect and **B, D** dorsal aspect. Carinae abbreviations: ventral prosubmedian (vps), ventral retrolateral (vrl), dorsal prolateral (dpl), prolateral median (plm), prolateral ventral (plv), dorsal retrolateral (drl), retrolateral median (rm), dorsal median (dm), ventral retrosubmedian (vrs), ventral median (vm), ventral prolateral (vpl), retrolateral dorsosubmedian (rlds). Scale bars: 10 mm.

**Figure 11.**
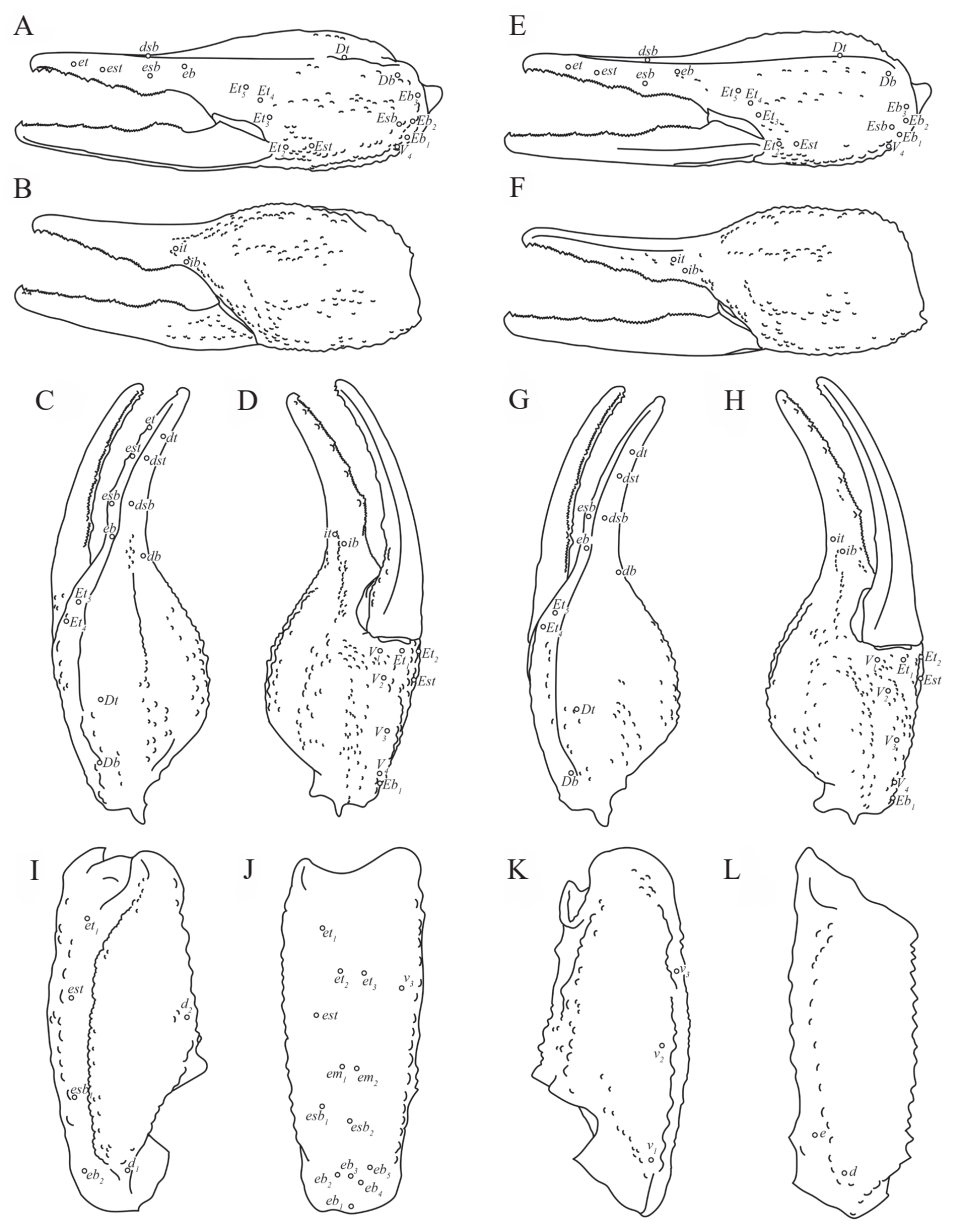
Illustrations of pedipalp of *Paruroctonustulare* sp. nov. from the type locality **A–D** chela, holotype male **E–H** chela, paratype female; **I–K** patella, holotype male; **L** femur, holotype male **A, E, J** retrolateral aspect **B, F** prolateral aspect **C, G, I, L** dorsal aspect **D, H, K** ventral aspect. Trichobothria indicated with open circles. Scale bars: 5 mm.

***Pedipalps*** (Figs 10, 11). Femur: Dorsal prolateral carina crenulate with two macrosetae on the proximal one-half; dorsal retrolateral carina weakly crenulate with two macrosetae on the proximal three-fourths. Dorsal surface finely granular. Retrolateral dorsosubmedian carina weakly crenulate with three large median macrosetae and a smaller one on the distal margin; retrolateral surface smooth aside from a few proximal granules. One large distal inframedial macroseta on the retrolateral surface. Three small ventral retrolateral macrosetae present, ventral surface with some irregular proximal granulation. Ventral prolateral carina crenulate. Prolateral surface irregularly and sparsely granular with three large prolateral ventral macrosetae on the proximal two-thirds, one prolateral ventrosubmedian macroseta near the midpoint, and a pair of macrosetae on the distal margin.

Patella: Dorsal retrolateral carina weakly crenulate with a proximal macroseta; dorsal prolateral carina crenulate with a proximal macroseta. Dorsal surface smooth. Retrolateral median carinae indistinct and very weakly crenulate, retrolateral surface otherwise smooth. A single median and two distal macrosetae are present on the retrolateral surface. Ventral retrosubmedian carina weakly crenulate with a distal macroseta; ventral prolateral carina crenulate with a distal macroseta, ventral median carina weakly crenulate. A proximal macroseta present at the junction of the ventral retrosubmedian and ventral median carinae. Ventral surface smooth. Prolateral median carina indistinct, represented by a few large granules, prolateral surface smooth with large proximal supramedian, proximal inframedian, distal inframedian, and distal supramedian macrosetae.

Chela: Dorsal prolateral carina appears as a dispersed field of granules on the manus with a medial macroseta, smooth on the fixed finger. Dorsal median carina weakly crenulate proximally and smooth distally, terminating at the base of the fixed finger with a single macroseta at its proximal extent. Dorsal retrolateral carina very weakly crenulate proximally and smooth distally, entirely smooth on the fixed finger, with proximal, medial, and distal macrosetae on the manus. Retrolateral median carina very weakly crenulate and indistinct with a medial macroseta. Ventral retrolateral carina irregular and weakly crenulate, with a proximal, medial, and 3/2 distal macrosetae. Intercarinal spaces on the dorsal and retrolateral surfaces smooth aside from occasional sparse granules. Ventral prosubmedian carina irregular and weakly crenulate, with proximal and medial macrosetae. Ventral prolateral carina irregularly crenulate to weakly crenulate with a proximal macroseta. Ventral surface mostly smooth with some distal granulation. Prolateral median carina irregularly crenulate to weakly crenulate with a proximal and medial macroseta. Internal surface mostly smooth with some weak, irregular granulation in the distal half and some fine granulation near the base of the fixed finger. The fingers are heavily scalloped, leaving a wide proximal gap when closed. The chela is uniformly finely granular at the base of this gap. Retrolaterally and prolaterally, the fingers are smooth except some fine proximal granulation. Macro and microsetae (22/19) are present on the ventral surface of the movable finger. One proximal retrolateral median, one proximal prolateral median, and one medial prolateral median macrosetae present on the movable finger. The fixed finger has one prolateral medial and one proximal dorsal prolateral, 1/0 retrolateral medial, and two distal dorsal retrolateral macrosetae. Both the fixed and movable fingers have five retrolateral enlarged denticles dividing the primary denticles into six sub-rows, with an additional retrolateral enlarged denticle at the distal extent of the movable finger, alongside the distal hook. On the fixed finger there are 5/6, 6/5, 8, 9, 12/10 denticles in primary rows I–V (40/38 total) and a terminal row (VI) with 14/13 denticles. On the movable finger there are 5/7, 8/9, 10/9, 11/10, 15/14 denticles in primary rows I–V (49/49 total) and a terminal row (VI) with 9/10. Each retrolateral enlarged denticle, as well as the distal fingertip hook, is accompanied by a single prolateral supernumerary denticle, for a total of six on the fixed finger and seven on the movable finger. There is a single macroseta posterior to each supernumerary denticle with the exception of the two distal-most on each finger. Two macrosetae are present proximal to the most proximal primary denticle on the fixed finger.

**Figure 12.**
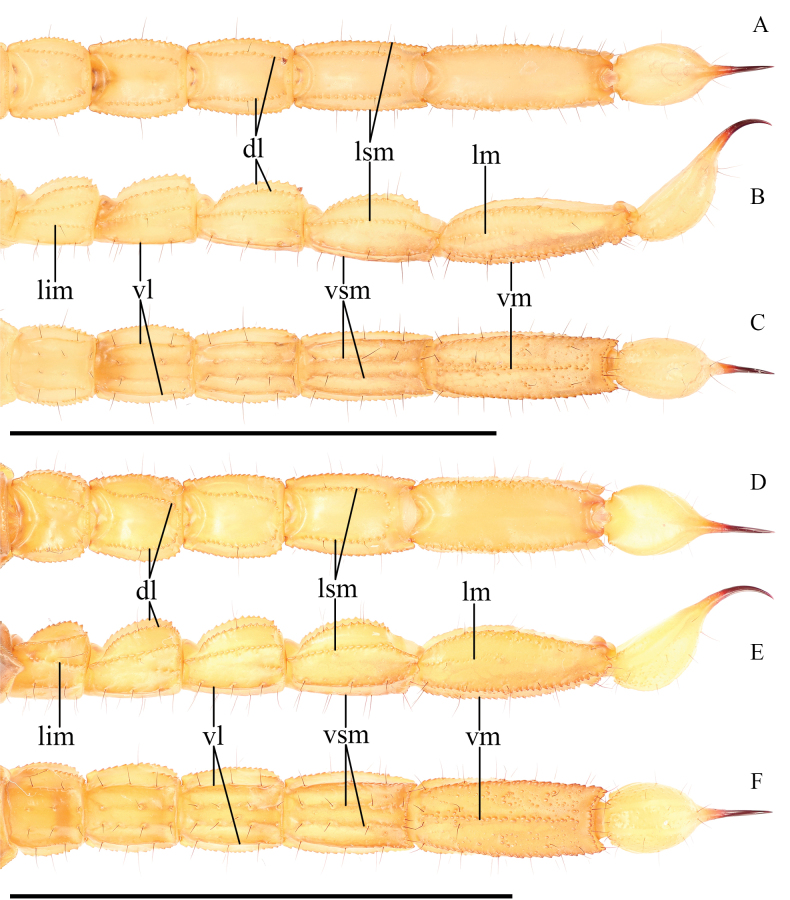
Metasoma of *Paruroctonustulare* sp. nov. from the type locality **A–C** holotype male and **D–F** paratype female **A, D** dorsal **B, E** lateral **C, F** ventral. Carinae abbreviations: dorsolateral (dl), lateral median (lm), lateral supramedian (lsm), lateral inframedian (lim), ventrolateral (vm), ventral submedian (vs), and ventromedian (vm). Scale bars: 10 mm.

***Metasoma*** (Fig. 12). Dorsal surface I–V smooth with occasional sparse granules. Dorsolateral carinae on segments I–IV strongly crenulate to serrate, weakly crenulate on V. Lateral surface smooth. Lateral supramedian carinae I–IV strongly crenulate to serrate. Lateral inframedian carinae crenulate on I–III, extend through only the posterior fifth or less of segments II-III. Lateral median carinae indistinct and weakly crenulate on V, extending proximal two-thirds. Ventrolateral carinae I–IV smooth, weakly crenulate on the posterior fourth of segment IV. Ventrolateral carinae on segment V strongly crenulate to serrate. Ventral surface of segment I–IV smooth; ventral surface sparsely granular on segment V. Ventral submedian carinae on I–IV smooth and unpigmented, indistinct on I. Ventromedian carina on segment V crenulate and irregular. Dorsolateral macrosetae I–V follow the pattern 0, 0, 1, 1, 3/4. Lateral supramedian macrosetae I–IV follow the pattern 0, 1, 1, 2. Two lateral median macroseta on V. Lateral inframedian macrosetae I–III follow the pattern 1, 0, 0. Ventrolateral macrosetae I–V, excluding any on the posterior margin of the segment, follow the pattern 2, 3/2, 3, 3. Six ventrolateral macrosetae on V, excluding any on the posterior margin. Ventral submedian macrosetae I–IV, excluding those on the posterior margin of the segment, follow the pattern 2, 2, 3, 3. 4/2 pairs of macrosetae are present between the ventromedian and ventrolateral carinae on segment V. Two pairs of macrosetae on the ventral posterior margin of metasomal segments IV–V; a single pair of macrosetae on the ventral posterior margins of other metasomal segments.

***Telson*** (Fig. 12). Very weakly ventro-anteriorly granular, otherwise smooth. Sparsely setose ventrally and laterally.

**Figure 13.**
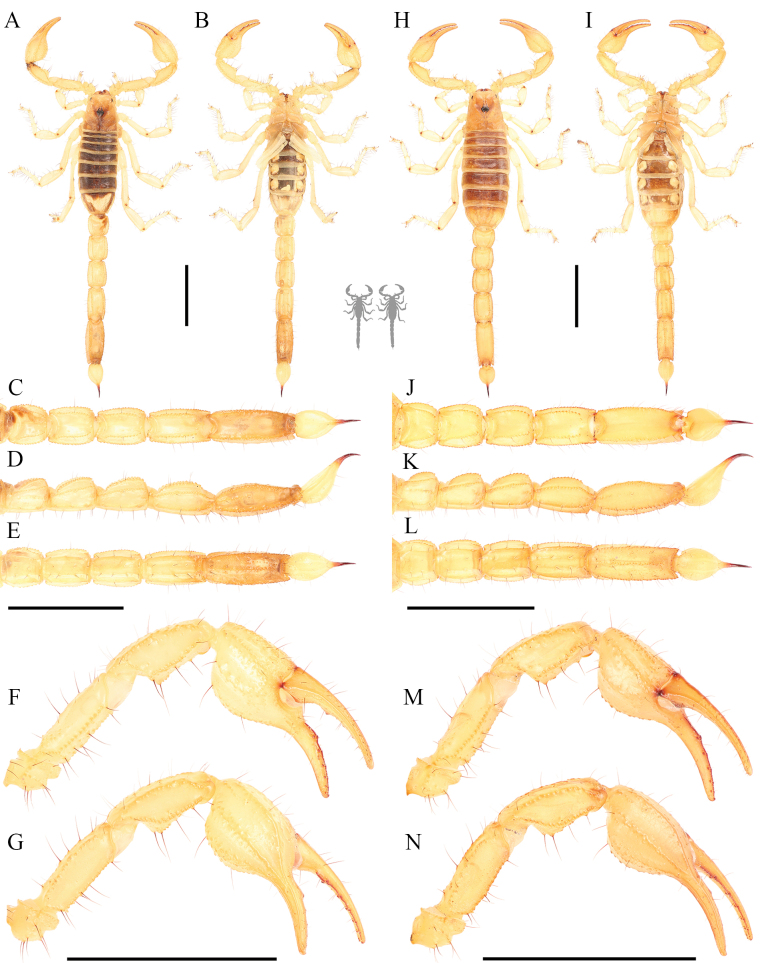
*Paruroctonustulare* sp. nov. from the northern locality N1 **A–G** adult male, **H–N** adult female **A, B, H, I** habitus; **C–E, J–L** metasoma **F, G, M, N** pedipalp **A, H, C, J, G, N** dorsal **B, I, E, L, F, M** ventral **D, K** lateral. Note pale body color and a total lack of fuscous patterning on the pedipalps and metasoma.Scale bars: 10 mm.

**Figure 14.**
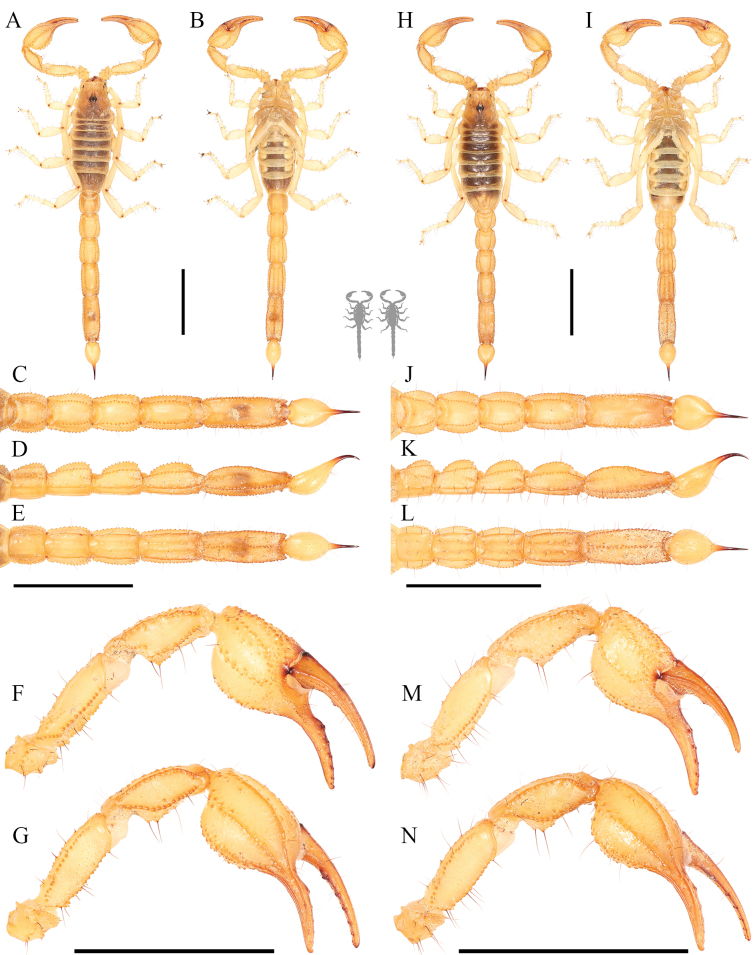
*Paruroctonustulare* sp. nov. from the southeastern locality SE1 **A–G** adult male, **H–N** adult female **A, B, H, I** habitus **C–E, J–L** metasoma **F, G, M, N** pedipalp **A, H, C, J, G, N** dorsal **B, I, E, L, F, M** ventral **D, K** lateral. Note dark body color and proportionally slightly more robust chelae than individuals from the northern and central localities. Scale bars: 10 mm.

**Female.** (Figs 6–8, 10–14) Sexual dimorphism is similar to that of many other *Paruroctonus* species. Coloration is more glossy, especially on the carapace and tergites. Carapace proportionally larger with slightly weaker granulation. Tergites with substantially weaker and sparser granules. Chela somewhat less incrassate and lacking scalloping in the fingers; closed fingers leave no significant gap. Proximal denticular row on the chelal fixed finger has substantially fewer denticles. Metasoma proportionally more robust than in males. Pectines smaller in both length and width with fewer teeth and slightly fewer and more regular middle lamellae. Sclerites of the genital operculum are slightly separated; genital papillae absent.

###### Variation.

(Figs 1, 13, 14, Suppl. material 3) Several differences exist between individuals from across this species’ range. Individuals from the northern locality appear to have no consistent differences from those from the central locality cluster aside from absent fuscous pigmentation on the ventral surface of the metasoma and pedipalps; this similarity matches the genetic similarity between the central cluster and northern localities. Several minor differences exist between individuals from the central localities and those from either southern locality. Individuals from both the southeastern and southwestern localities are generally darker and more orange with no fuscous markings on the ventral surface of the metasoma or on the pedipalps. Male chelae are more incrassate and the scalloping on the fixed finger is significantly more prominent. However, despite some genetic divergence, individuals from the southeastern and southwestern localities have no consistent morphological differences from one another.

Coloration: Coloration fairly variable, ranging from lighter yellows and tans to darker oranges and browns. Extents of fuscous pigmentation also fairly variable with at least some of the variation being correlated with locality of origin. In individuals from the central localities, fuscousity on the interocular region of the carapace almost always present but fairly variable in scope along with fuscousity on the ventral surface of the metasoma (along the ventral submedian keels). Fuscousity on the pedipalps ranges from faint to absent.

Carapace: Granulations essentially consistent within each sex. Larger granules posterior to the median eyes, decreasing in size anteriorly and laterally.

Mesosoma: Granulations essentially consistent within each sex. Largest and densest granulation on the posterior-lateral corners of each tergite.

Pectines: Variable numbers of teeth and lamellae: males, 23–29 teeth and 16–28 middle lamellae; females, 17–20 teeth and 13–19 middle lamellae.

Legs: Setation on the basitarsi largely variable, setation on the telotarsi relatively consistent. Two primary basitarsal retrosuperior spinoid setae consistent, others occasionally present. Distal prosuperior and superior accessory macrosetae typically present. Any other important variation in setation summarized in Suppl. material 3.

***Taxonomically useful characters*.** Selected characteristics of *Paruroctonustulare* sp. nov. are presented here based on how taxonomically useful they are when comparing P.tulare sp. nov. to similar *Paruroctonus* species. Most items in the below lists are applicable to other alkali-sink *Paruroctonus* species as well.

1. Setal counts on the lateral and ventral surface of the metasoma are consistent and differ from those of several similar species.
2. Setal counts on the pedipalp patella are consistent and differ from those of several similar species.
3. Setal counts on the manus (aside from the ventral surface) are consistent and differ from those of several similar species.
4. Morphometric ratios of metasomal segments differ from those of several similar species.
5. Coloration is quite variable but fuscousity is consistently isolated to specific regions and thus differs from certain similar species.
6. Setation on the telotarsi is somewhat variable but differs from that of certain similar species.
7. Morphometric ratios of manus size are somewhat variable but are diagnostically useful from certain similar species.
8. The number of denticles on certain carinae on the pedipalp patella and femur are substantially different than on certain similar species.

***Less useful characters*.**

1. Setation on the chelal fingers is variable and is not substantially different from that of most similar species.
2. The number of primary denticles on the chelal fingers is variable and not substantially different than on most similar species.
3. The number of pectinal teeth is variable and not substantially different than on most similar species.
4. Setation on the ventral surface of the manus is variable and is not substantially different from that of most similar species.
5. Setation on the dorsal surface of the metasoma is variable and not substantially different than on most similar species.
6. Setation on the basitarsi is both variable and irregular leading to ambiguity in nomenclature and is not diagnostically useful against most similar species.
7. Denticle counts on carinae on the manus are variable and often ambiguous and thus not diagnostically useful.

###### Habitat, distribution, ecological notes, and IUCN assessment.

*Paruroctonustulare* sp. nov. is only known from lowland, alkali-sink habitats in the Tulare Basin (Fig. 2), a large and typically endorheic watershed which formerly contained Tulare Lake at its center (ECORP 2007). Its known distribution can be divided into into a northern locality (N1) in Fresno county, a central locality cluster (C1–9) in northern Kern county, a southeastern locality (SE1) in southern Kern county, and a southwestern locality (SW1) in southern Kern county (Fig. 2). The northern and central cluster localities represent a population while the two southern localities represent two additional populations.

**Figure 15.**
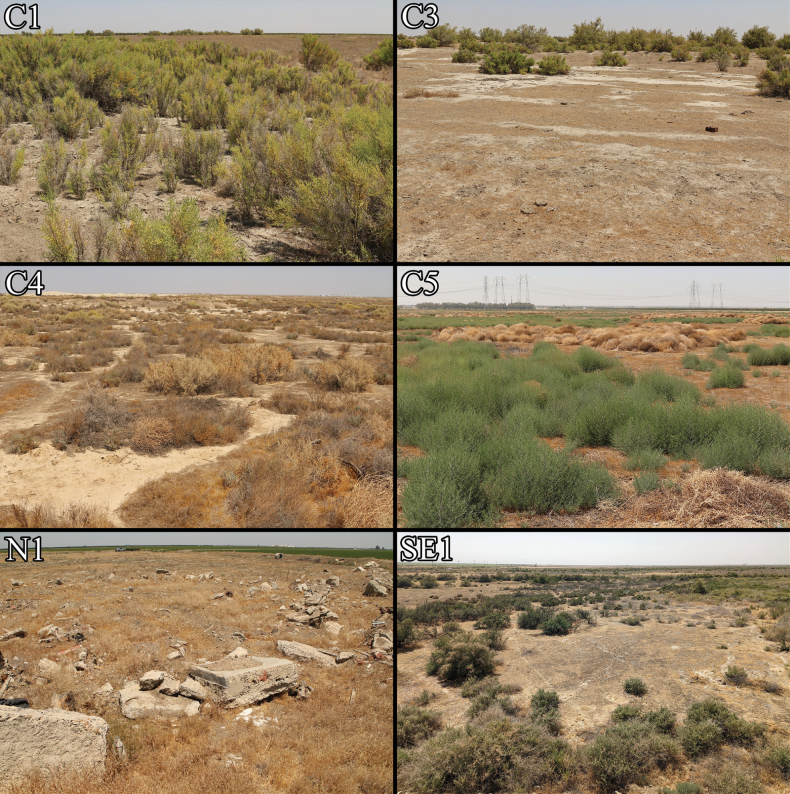
Habitat of *Paruroctonustulare* sp. nov. Locality codes correspond to those in Table 1. Photographs of C1–5 taken in August 2021, N1 taken in May 2021, SE1 taken in May 2022.

***Distribution and habitat*** (Figs 2, 15). **Central localities**: The central locality cluster contains nine localities, including the type locality, encompassing a convex polygon of approximately 193 km^2^ in northern Kern County. Notably, this area includes significant quantities of unsuitable habitat including scrubland, invasive grassland, perennial marshland, and developed agricultural land (Dewitz and USGS 2021). Outside this polygon, *P.tulare* sp. nov. may be present in additional suitable alkali-sink environments, primarily to the north, a hypothesis that our distribution model supports. Any additional habitat in this area is likely to be heavily restricted as this area has been mostly converted for agriculture or urban development (Dewitz and USGS 2021). In total, excluding areas that are permanently flooded or have been converted for agriculture, distribution models suggest ~ 134 km^2^ of suitable habitat remains in this area. Of the central cluster localities, two, including the type locality (Fig. 15) (localities C1, the type locality, and C2), consist of relatively pristine habitat, dominated by native *Allenrolfeaoccidentalis* and small native herbs such as *Suaedanigra*, *Frankeniasalina*, *and Cressatruxillensis*. The dominant grass is *Distichlisspicata*, with occasional patches of other grasses of uncertain origin or identity; however, much of the ground remains as bare soil.

The central locality C8 is also relatively pristine with mostly native plants including a limited number of *A.occidentalis* surrounded by *S.nigra*, *Isocomaacradenia*, *Atriplexlentiformis*, and sparse small grasses of uncertain origin or identity. One individual of *P.tulare* sp. nov. was posted by Galen Freed-Wilhelm on iNaturalist in March of 2022 within the confines of Kern National Wildlife refuge (C9) in what appears to be fairly high-quality habitat, although it is limited in scope by the water bodies on either side. An additional locality (C7) is dominated by *A.occidentalis* with patches of short grass of uncertain identity or origin; however, the aforementioned native herbs and *D.spicata* are absent. Three additional localities (C3, C5, C6) are more disturbed: C3, just south of localities C1 and C2, has seen significant damage from cattle overgrazing; native herbs and *D.spicata* are absent and *A.occidentalis* is somewhat reduced in density. Introduced and ecologically detrimental *Tamarix* sp. are present in large numbers (Hamada et al. 2007). Locality C5 is heavily dominated by tall, presumably introduced grass species and the invasive and ecologically detrimental plant *Salsolatragus* (Smith 2005). Locality C6 is immediately adjacent to riparian/wetland habitat and is relatively less damaged than C3 and C5, but *A.occidentalis* and the aforementioned native herbs are still not present, instead replaced by large *I.acradenia* and *Atriplexpolycarpa* shrubs and grasses of uncertain origin. Non-native *Tamarix* sp. are present, especially in the adjacent riparian area. The final locality (C4) is distinctly different from the others: it appears to be a transition from alkali-sink to scrubland, dominated by *Atriplexspinifera*, *A.polycarpa*, and *S.nigra*, with *A.occidentalis* entirely absent. Unlike any of the aforementioned localities, the dominant *Paruroctonus* is *P.variabilis*, which was found with fairly high frequency. Only a single *P.tulare* sp. nov. could be found and was within a few meters of an individual of *P.variabilis*. Other scorpion species recorded in sympatry with *P.tulare* sp. nov. in this region were: *Hadrurusobscurus* at locality C4 (recorded on iNaturalist), *Serradigitus* sp. at C3 and C7, and *Paravaejovis* sp. at C1, C3, C4, and C7.

In our sampling of the central localities by UV light on August 9, 2021, *P.tulare* sp. nov. was found to be very abundant at localities C1 and C2, less common at locality C6, uncommon at localities C3, C5, and C7, and very tough to find at locality C4. In additional sampling by UV light on April 4, 2022, *P.tulare* sp. nov. was fairly common at locality C8. While it is not possible to make high-confidence conclusions about population size and absence with only a single night of sampling at each locality, it appears that *P.tulare* sp. nov. is able to achieve a high population density in high-quality habitat. There appears to be a correlation between the population density of *P.tulare* sp. nov. and habitat purity in areas where it is the dominant *Paruroctonus* species.

**Northern locality**: The northern locality (N1) constitutes two small patches of land separated by a two-lane road. These two small patches have an approximate area of 1.9 and 1.6 hectares respectively and are entirely surrounded by agricultural development. While this locality is heavily disturbed and is littered with concrete and other discarded debris, *P.tulare* sp. nov. continues to persist.

It is difficult to know the original habitat of northern locality N1 due to the extensive agricultural development and land disturbance in the area. Common plant species consist of introduced generalist species (*Salsolatragus*, *Malva* sp., *Latucaserriola*, and various introduced grasses and mustards), native alkaline specialist species (*Suaedanigra*, *Distichlisspicata*, *Atriplexlentiformis*, and *Frankeniasalina*), and a few miscellaneous species (native *Helianthusannuus*, *Asclepiasfascicularis*, and *Heliotropiumcurassavicum*; non-native *Arundodonax*). A largely alkali-sink specialist species, *Suaedanigra*, dominates the area, especially during summer months. This combination of plants and especially the presence of *S.nigra*, *D.spicata*, and *F.salina*, all of which were also found at the type locality, suggests that the original habitat at the northern locality likely was also an alkali-sink. The clay-rich soil with a pH of 8.2 further backs this up (Poggio et al. 2021). Notably, *Allenrolfeaoccidentalis* was not found at N1, even though it was the dominant plant species in several of the central as well as the southeastern and southwestern localities. This, however, can be explained by the frequent habitat clearing in portions of the northern locality, effectively preventing the growth of larger perennial plants.

At N1, the authors found *P.tulare* sp. nov. underneath slabs of concrete (March, April, May) or found them by UV light at night (May, July). Each of the seven surveys conducted by the authors, Brian Hinds, and Noah Morales at N1 from March to May involving flipping and/or backlighting detected only 0–4 individuals. Searching by UV light during this period proved especially ineffective, as in three attempts only a single adult female was found. However, fifteen individuals were found in a single UV light survey in July 2021, including the only adult males found at N1. This suggests that the surface activity of this species in the heavily disturbed northern locality may be very sporadic. If that is the case, possible explanations include escaping habitat clearing that takes place in spring or avoiding flooding during the rainy season. The highly unusual state of N1 makes it difficult to make any conclusions about the activity at other localities based on the activity at N1 and vice versa. However, frequent habitat clearance at N1 likely causes heavy stress to the habitat and likely is causing the population there to be in decline.

No scorpion species have been found in sympatry with *Paruroctonustulare* sp. nov. at N1. The geographically closest *Paruroctonus* species is *Paruroctonusvariabilis* (the nearest record is near Mendota Wildlife Area at a distance of ~ 14 km). On a night when *P.tulare* sp. nov. was successfully found at N1, surveys of eight other small patches of habitat within 20 km of the north plot failed to locate scorpions of any species. The habitat conditions at these localities ranged from heavily disturbed to similar or slightly better than N1 where *P.tulare* sp. nov. has been confirmed. As *P.tulare* sp. nov. can be very unreliable to find at N1, a lack of specimens found while surveying does not necessarily imply that they are absent at all of these aforementioned locations, and we urge additional surveying to locate possible alternate localities.

**Southeastern and southwestern localities**: The southeastern and southwestern localities represent independent populations. The southeastern (SE1), is a small area of alkali-sink habitat adjacent to a more extensive wetland region at the southern shoreline of the historical Kern Lake. Individuals of *P.tulare* sp. nov. were concentrated in approximately one hectare of habitat but a small number were found within the same plot of land to the northwest. The alkali-sink plant community in this area appears to be somewhat intact, including patches of *A.occidentalis* and *S.nigra* surrounded by *A.lentiformis*, A.cf.polycarpa, *I.acradenia*, and sparse small grasses of uncertain identity or origin. The southwestern locality (SW1) is located approximately 19 km to the northwest, and is a small isolated patch of alkali-sink habitat near what was historically the southern shore of Buena Vista Lake. Members of an alkali-sink plant community such as *S.nigra* and A.cf.polycarpa are present in high densities.

In our sampling of SE1 by UV light on April 5, 2022, *P.tulare* sp. nov. was found to be fairly abundant; adult females and juveniles were numerous but only a single adult male was found. In our sampling of SE1 by UV light on September 17, 2022, *P.tulare* sp. nov. was found to be fairly common; adult males were numerous and a few juveniles were found but only a single adult female was observed. In our sampling of SW1 by UV light on September 17, 2022, *P.tulare* sp. nov. was found to be abundant; adult males were very numerous and many juveniles and adult females were observed. We recorded *Paravaejovis* sp. at SW1; no other scorpion species were observed at either locality within the southern range.

**Absence.** Surveys at two additional locations, both approximately halfway between the northern and central ranges of *P.tulare* sp. nov., unsuccessful. No scorpions were found at the first: an open tamarisk woodland and grassland area north of Huron (36.2443, -120.1028). The habitat at this spot is different from the habitat at any locality where *P.tulare* sp. nov. has been recorded and does not resemble an alkali-sink, so the absence of *P.tulare* sp. nov. is expected.

The second is an area of alkali-sink habitat near Lemoore (36.2430,-119.8112) largely dominated by *Allenrolfeaoccidentalis* and surrounded by drier habitat dominated by grasses of uncertain identity or origin. Although this habitat closely resembles the type locality of *P.tulare* sp. nov., none were found. *Paruroctonusvariabilis*, however, was found to be abundant in the grassland habitat and somewhat common in the alkali-sink habitat. It is unclear why *P.tulare* sp. nov. was not found during our survey of this area.

**Figure 16.**
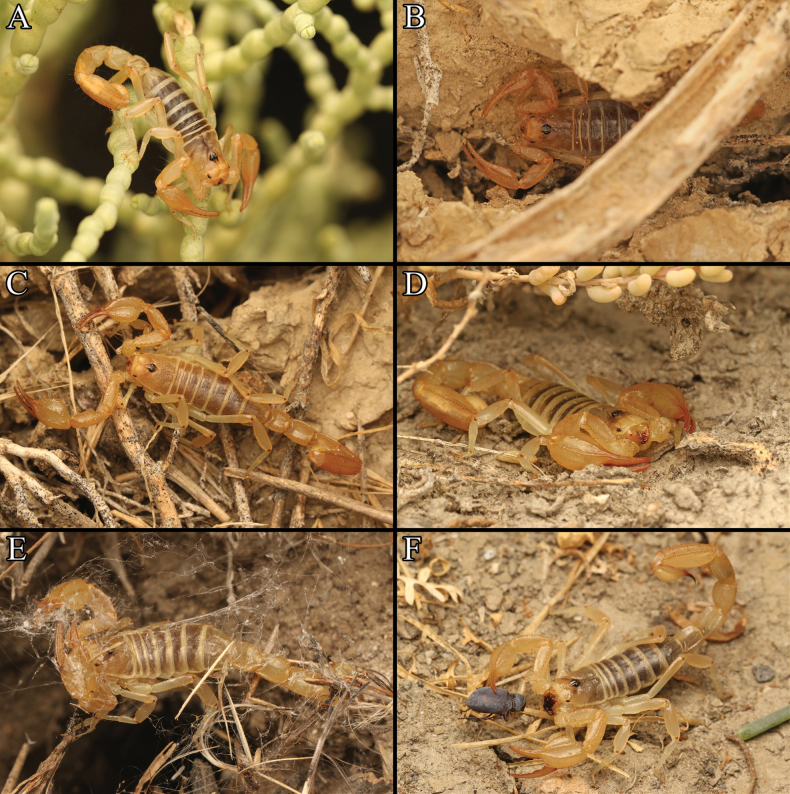
In-situ images of *Paruroctonustulare* sp. nov. of various life stages depicting behavior typical of the species: **A** juveniles perched on vegetation or **B** utilizing soil cracks for shelter **C** adult male wandering **D** adult female sheltering under vegetation. Images of intraspecific interactions involving *P.tulare* sp. nov. **E** predation by *Latrodectushesperus***F** predation on *Triorophus* sp.

**Behavior** (Fig. 16). *P.tulare* sp. nov. has been observed feeding on various small invertebrate species in the wild including solifuges (*Eremobates* sp.), beetles (*Triorophus* sp.), and cockroaches (*Arenivaga* sp.). This implies that they are generalist predators, as is the case in other Smeringurinae (Polis 1980). A single instance of predation on *P.tulare* sp. nov. by *Latrodectushesperus* was observed.

**Figure 17.**
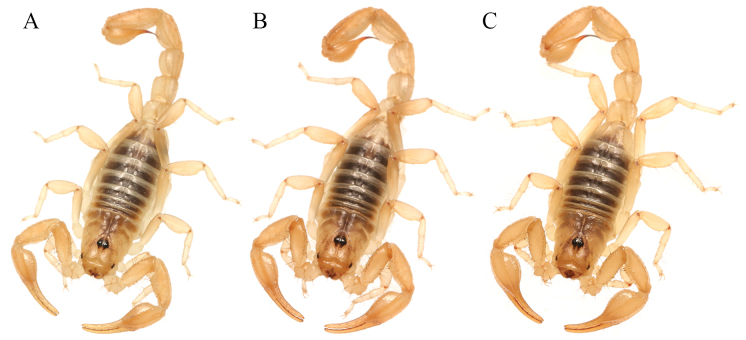
Immature individuals of *Paruroctonustulare* sp. nov. from the **A** central localities and **B, C** southeastern/southwestern localities.

In early August (localities C1 and C2) and mid-September (localities SE1 and SW1) sampling, putative intraspecific niche partitioning was observed between juvenile (Fig. 17) and adult individuals. Juveniles, especially smaller individuals, were typically found several centimeters up on plants, typically facing downwards. They were found most frequently on *Suaedanigra* and *Frankeniasalina*; however, individuals were occasionally found on *Allenrolfeaoccidentalis* and *Cressatruxillensis*. In contrast, adults and late-instar juveniles at all localities were almost exclusively found directly on the surface of the soil, often adjacent to burrows or underneath *Allenrolfeaoccidentalis* or *Suaedanigra* plants. In both cases, individuals appeared to be engaging in sit-and-wait predation, as several individuals were observed actively eating in both positions. This appears to be an important behavior for their survival, as in central localities where *S.nigra* and *F.salina* were absent, the authors found few or no early-instar juveniles, even though they outnumbered adults in surface-activity at localities C1 and C2.

In early April sampling (C8 and SE1), *P.tulare* sp. nov. were almost exclusively found in cracks in the soft clay soil with only a small number of individuals exposed on the surface of the soil and a single juvenile on a plant, possibly to avoid predation or exposure. It is unclear why certain individuals were active on the surface as none were observed actively feeding or in a typical predatory position. Similar patterns of activity were observed among females in early August and mid-September sampling (localities C1–7, SE1, SW1), suggesting that females are less prone to surface movement than males or that the activity period for females may be different than that for males.

Burrows of *P.tulare* sp. nov. were observed on multiple occasions (localities N1, C1, C2, C7, C8, SE1, and SW1). In each case, burrows were located in soft clay soil and typically in a small patch free of plants. In a few instances at the northern locality, burrows were associated with discarded concrete or tires. Natural cover objects for shelter such as rocks or logs are absent from known localities of *P.tulare* sp. nov. Therefore, we hypothesize that *P.tulare* sp. nov. have to create deep burrows in the soft clay soil or shelter deep in cracks in the clay for protection from the extreme daytime summer heat throughout their range. If this is the case, open soft clay soil free of vegetation is critical for their survival.

**Figure 18.**
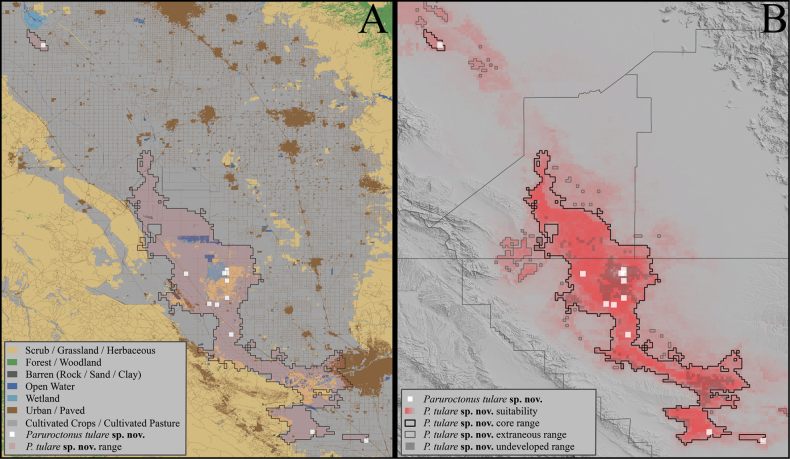
**A** Predicted historic distribution of *Paruroctonustulare* sp. nov. using Maximum Entropy distribution models overlaid on a land-usage map showing the conversion of the majority of the historic distribution of *P.tulare* sp. nov. to cropland and urban land **B** maximum entropy niche models indicating the hypothesized historic and current distribution of *P.tulare* sp. nov.

**Conservation** (Figs 15, 18). *Paruroctonustulare* sp. nov. experiences a wide variety of imminent threats to its survival including, but not limited to, habitat destruction due to agricultural development and urbanization, overuse of water and degradation of wetland areas, the presence of ecologically detrimental non-native species, and climate change. Likely the most significant threat to *P.tulare* sp. nov. has been land conversion for agriculture and urban development. While data on the original extent of alkali-sink habitat in the San Joaquin Valley is limited, wetland habitats and wetland-adjacent habitats (including alkali-sinks) in the San Joaquin Valley have declined by approximately 90% compared to pre-European levels (Kelly et al. 2005). Our distribution model indicates that suitable habitat for P.tulare sp. nov. historically encompassed ~ 2194 km^2^, but 85% of this area has been developed for agriculture or urbanism, resulting in only 336 km^2^ of remaining suitable habitat (Fig. 18). The true extent of occupied habitat is likely substantially lower with the largest contiguous portion of suitable undeveloped habitat containing *P.tulare* sp. nov. occurrence records being only 134 km^2^ in size, including areas that have been degraded by factors such as grazing. We estimate that the overall reduction in potential habitat for *P.tulare* sp. nov. is nearly 94% of the original suitable range.

Fortunately, conversion of new wetland/alkali-sink habitat has slowed since ~ 1980 (Kelly et al. 2005). In the coming decades, over 2,000 km^2^ of once-farmed land are projected to be abandoned due to insufficient water resources, climate change, and other factors (Stewart et al. 2019). Much of this land can be restored to a more natural state, facilitating the recovery of many endangered species (Stewart et al. 2019). Large quantities of land with potential for restoration exist near to the central and southeastern/southwestern localities of *P.tulare* sp. nov. but have not yet been considered for restorative action as current efforts have largely focused on arid-adapted species (Stewart et al. 2019). We urge that some of this land be placed into conservation in order to restore the rare alkali-sink habitats of the San Joaquin Valley where *P.tulare* sp. nov. and several other endemic species can survive.

Of the 12 localities where *P.tulare* sp. nov. has been recorded, (nine in the central cluster, as well as the northern, southeastern, and southwestern localities), only five (C1, C2, C9, SE1, SW1) are relatively large in area and pristine, with mostly native plants and little to no damage from agriculture, dumping, or livestock. The northern locality (N1) is almost entirely destroyed by dumping and frequent plant removal, the central localities C3 and C5 are close to unsuitable due to a lack of native plants and an abundance of non-native plants respectively. In areas with the heaviest non-native plant cover or least native plant cover, *P.tulare* sp. nov. was not observed.

We hypothesize that invasive European grasses negatively impact *P.tulare* sp. nov. in a number of ways: *P.tulare* sp. nov. juveniles appear to rely on small forbs while hunting for food. As these plants are replaced by dense and flimsy grasses, juveniles cannot use them effectively. *Paruroctonustulare* sp. nov. appears to rely on creating burrows or sheltering in clay cracks to escape the daytime heat. Non-native grasses produce long, shallow roots early in the growing season (Phillips et al. 2019), which are likely to impede the burrowing ability of *P.tulare* sp. nov. during the warmer months. Thick non-native grasses are likely to impede the locomotion and sensory abilities of *P.tulare* sp. nov., causing difficulty in hunting for prey and searching for mates.

Livestock grazing may also threaten the survival of *P.tulare* sp. nov. Cattle grazing has been found to have effects on native desert species (Dobkin et al. 1998; Marty 2005; Germano et al. 2012). In some cases in California’s Central Valley, livestock grazing has been determined to have a beneficial effect on certain native animal species, mostly by reducing dominant non-native grasses and therefore increasing vernal pool water retention, reducing fire risk, and improving animal locomotion (Marty 2005; Germano et al. 2012). However, in areas where non-native grasses are not dominant, cattle grazing has been shown to facilitate the establishment and spread of non-native grasses, have a disproportionate negative impact on native perennial forb species, and generally decrease biodiversity, especially in arid areas (Dobkin et al. 1998; Kimball and Schiffman 2003; Hayes and Holl 2003; Germano et al. 2012). We hypothesize that a decline in native herbs such as *Suaedanigra*, *Frankeniasalina*, and *Cressatruxillensis* (due to livestock grazing) will have a negative effect on *P.tulare* sp. nov. as juveniles appear to rely on them and there is the potential for soil impaction that could impact the construction or maintenance of burrows in soft clay soil. Possible evidence of this effect was observed at locality C3, where heavy cattle grazing has occurred: *P.tulare* sp. nov. was relatively difficult to find, and no early instar juveniles were observed. This is in contrast to observations at proximal localities C1 and C2. We recommend further research be done to assess these effects, and strongly urge that cattle grazing be discontinued in alkali-sink areas as it is likely to cause local extinctions of native plant and animal species and exacerbate the problem of non-native grass establishment.

Currently, very little of the known range of *P.tulare* sp. nov. exists within protected areas. A small amount of land near localities C2 and C4 is owned by private conservation groups; however, the former has damage from livestock grazing and the latter is marginally suitable for *P.tulare* sp. nov. The species has been observed in some portions of Kern National Wildlife Refuge, and some native plants are present at Unit 15 and some of the western half of the refuge, indicating alkali-sink habitats (Cypher et al. 2012). Aside from these, there are no protected areas where we have high confidence of *P.tulare* sp. nov. occurrence, so protecting known localities should be a priority. In summary, *P.tulare* sp. nov. faces a large number of imminent threats to its survival, most of them anthropogenic in nature. The most important step towards the conservation of *P.tulare* sp. nov. is the preservation of alkali-sink plant communities by protecting the remaining high-quality habitat, controlling invasive species, limiting cattle grazing, restoring abandoned land, and combating the causes and effects of climate change. Furthermore, due to its small and unstable range, we suggest that this species receive endangered or critically endangered species status, at least at the state level, so that it can be adequately protected.

**Additional risks.** Aside from European invasive grasses, two additional other non-native species frequently observed present in the range of *P.tulare* sp. nov. are *Tamarixramosissima* and *Salsolatragus*. The former is a large shrub or tree which grows in riparian or wetland areas and causes significant ecological degradation by using large quantities of water, something that may negatively affect alkali-sink habitats, as they are closely associated with wetlands (Hamada et al. 2007). The latter is a non-native salt-tolerant weed which threatens alkali-sink environments due to its high flammability and ability to outcompete native plants (Smith 2005), as can be seen at the central locality C5, where it almost completely covers the landscape. Furthermore, we urge that steps be taken to replenish native small plant populations before these areas are overtaken by invasive plant species, which, in many cases, can outcompete native plants during the growing season (Phillips et al. 2019). In areas where *P.tulare* sp. nov. is absent and non-native grasses cover the habitat, simply grazing the area to control these grasses is likely to be insufficient for restoring *P.tulare* sp. nov. populations, so recovery plans must take the possible negative effects of cattle grazing on these alkali-sink ecosystems into account and work towards restoring a more natural ecosystem. This will likely also include controlling other non-native species such as *Tamarixramosissima* and *Salsolatragus*.

Pesticide usage in California’s Central Valley has been identified as a major cause of decline in certain insect species, especially those that are small-bodied, even in areas where pesticides are not directly applied (Forister et al. 2016). As all known localities for *P.tulare* sp. nov. are in relatively close proximity to farmland, the effects of pesticides on their habitats may be significant. While it is unclear to what degree various pesticides affect scorpions, widespread neonicotinoid insecticide use is very likely to negatively affect the populations of prey items (Forister et al. 2016).

In the future, climate change is likely to present an ever-increasing threat to Tulare Basin ecosystems (Fernandez-Bou et al. 2021). Temperature increases and decreases in water resources in the San Joaquin Valley will directly negatively impact wetland regions (Fernandez-Bou et al. 2021) and their associated alkali-sinks. With further stresses to agricultural production and substantial population increases projected by mid-century, it is likely that these already scarce water resources will become even less available to natural ecosystems (Fernandez-Bou et al. 2021). Historically, decreases in precipitation in California’s Mojave Desert have correlated with lower lake levels and habitat contraction for alkali-sink adapted species (Stoffer 2004). As lake levels in the Tulare Basin have closely matched those in the Mojave Desert during the Holocene (Negrini et al. 2006), similar effects on alkali-sink specialist species such as *P.tulare* sp. nov. are likely to occur. With the San Joaquin Valley’s wetlands and alkali-sink environments already in such a diminished state, reduced precipitation and increased evaporation rates may be an existential threat to *P.tulare* sp. nov. Renewable energy sources are some of the strongest tools to combat climate change, and the San Joaquin Valley has high potential for solar energy generation (Phillips and Cypher 2019). This is especially true for some central alkali-sink habitats, which are flat and receive a high degree of solar radiation (Phillips and Cypher 2019). However, these areas also have a large number of endangered species, and it is important not to jeopardize their conservation efforts when constructing solar facilities. While current estimates of habitat value have identified arid areas in the San Joaquin Desert where habitat preservation or restoration is more important than solar energy generation (Phillips and Cypher 2019), we believe they have not sufficiently considered the importance of preserving and restoring the alkali-sink environments in the San Joaquin Valley, listing them as largely low-value land. We suggest that habitat values be reevaluated taking *P.tulare* sp. nov. into account to maximize the preservation of endangered alkali-sink habitats.

***IUCN Red-List assessment*.** Based on IUCN Mapping standards (IUCN 2022), the known distribution of *P.tulare* sp. nov. has an Extent of Occurrence (EOO) of 1855 km^2^ while the projected distribution has an EOO of 5720 km^2^. The known Area of Occupancy (AOO) (IUCN 2022) of *P.tulare* sp. nov. covers 44 km^2^, although distribution models of undeveloped contiguous suitable habitat suggest that the AOO may be up to 168 km^2^. The historic AOO of *P.tulare* sp. nov., when scaled to the IUCN mapping standard of 4 km^2^ grid cells (IUCN 2022), covers 2196 km^2^, however, most of this land is no longer suitable for *P.tulare* sp. nov.

The varied threats faced by *P.tulare* sp. nov. include some that may have effects on a local scale (such as invasive species) and others on a regional scale (such as climate change) meaning that the number of IUCN standard locations (IUCN 2022) is highly dependent on the primary threat identified. Here we recognize threats to habitat quality, something that depends on most major threats to *P.tulare* sp. nov., as the most important factor in designating IUCN locations. While some threats (such as climate change) may affect all localities at once, using a combination of different threats, we consider the 12 known localities of *P.tulare* sp. nov. to best represent five IUCN locations based on current habitat contiguity: N1; C1–7 and C9; C8; SE1; and SW1.

Continuing decline for *P.tulare* sp. nov. has been inferred based on the numerous threats to the species outlined under Conservation. This decline most severely affects:

- Extent of occurrence if N1 becomes extinct
- Area of occupancy if grazing continues to degrade the habitat at central localities.
- Area, extent, and/or quality of habitat if grazing, climate change, or pesticide usage continue to degrade habitats
- Number of locations or subpopulations if N1 becomes extinct
- Number of mature individuals if grazing affects reproduction rates

Therefore, this species reaches the criteria to be listed by the IUCN as Endangered with a red-list assessment of **EN B1ab (i, ii, iii, iv, v) + 2b (i, ii, iii, iv, v)**. It is possible that *P.tulare* sp. nov. could qualify for endangered or vulnerable species status through criteria A3 or A4 as the aforementioned decline factors have the potential to cause a > 30% population decrease during upcoming decades; however, as the generation time for Vaejovid scorpions is entirely unknown, we have no way of making a good estimate of the length of three generations for *P.tulare* sp. nov. (IUCN 2012).

###### Etymology.

The specific epithet *tulare* refers to the Tulare Basin, the region to which *Paruroctonustulare* sp. nov. is endemic.

## ﻿Discussion

*Paruroctonustulare* sp. nov. is the fourth known species of alkali-sink *Paruroctonus* from the Mojave and San Joaquin desert region. Morphological similarities between *P.tulare* sp. nov. and other alkali-sink scorpions, especially between *P.tulare* sp. nov. and *P.conclusus*, suggest that the alkali-sink *Paruroctonus* species may share common ancestry (Jain et al. 2022). Further genetic work will be necessary to determine the relationship between this species and other alkali-sink *Paruroctonus* and the details of its diversification.

## Supplementary Material

XML Treatment for Paruroctonus tulare

## ﻿Additional information

### Author ORCIDs

Jacob A. Gorneau https://orcid.org/0000-0001-9230-9774

Lauren A. Esposito https://orcid.org/0000-0002-5514-7486

### Data availability

The input and output files underpinning the analyses reported in this paper are deposited in in Zenoodo at https://doi.org/10.5281/zenodo.8408838. The data underpinning the analysis reported in this paper are deposited at GBIF, the Global Biodiversity Information Facility, and are available at https://ipt.pensoft.net/resource?r=paruroctonus_tulare.

## Supplementary materials

Supplementary material 1 GenBank accession numbers for specimens for which cytochrome oxidase c subunit I (COI) was sequenced for use in the haplotype network Data type csv https://binary.pensoft.net/file/944629This dataset is made available under the Open Database License (http://opendatacommons.org/licenses/odbl/1.0/). The Open Database License (ODbL) is a license agreement intended to allow users to freely share, modify, and use this Dataset while maintaining this same freedom for others, provided that the original source and author(s) are credited.Prakrit Jain, Harper Forbes, Jacob A. Gorneau, Lauren A. Esposito

Supplementary material 2 Complete list of records used to create map in Fig. 5 Data type xlsx https://binary.pensoft.net/file/944630This dataset is made available under the Open Database License (http://opendatacommons.org/licenses/odbl/1.0/). The Open Database License (ODbL) is a license agreement intended to allow users to freely share, modify, and use this Dataset while maintaining this same freedom for others, provided that the original source and author(s) are credited.Prakrit Jain, Harper Forbes, Jacob A. Gorneau, Lauren A. Esposito

Supplementary material 3 Variation in selected morphological characters across *Paruroctonustulare* sp. nov. specimens examined in this study Data type xlsx https://binary.pensoft.net/file/944631This dataset is made available under the Open Database License (http://opendatacommons.org/licenses/odbl/1.0/). The Open Database License (ODbL) is a license agreement intended to allow users to freely share, modify, and use this Dataset while maintaining this same freedom for others, provided that the original source and author(s) are credited.Prakrit Jain, Harper Forbes, Jacob A. Gorneau, Lauren A. Esposito
